# A New Membrane Protein Sbg1 Links the Contractile Ring Apparatus and Septum Synthesis Machinery in Fission Yeast

**DOI:** 10.1371/journal.pgen.1006383

**Published:** 2016-10-17

**Authors:** Kriti Sethi, Saravanan Palani, Juan C. G. Cortés, Mamiko Sato, Mayalagu Sevugan, Mariona Ramos, Shruthi Vijaykumar, Masako Osumi, Naweed I. Naqvi, Juan Carlos Ribas, Mohan Balasubramanian

**Affiliations:** 1 Temasek Life Sciences Laboratory, National University of Singapore, 1 Research Link, Singapore; 2 Department of Biological Sciences, National University of Singapore, Singapore; 3 Division of Biomedical Sciences, Warwick Medical School, University of Warwick, Coventry, United Kingdom; 4 Instituto de Biología Funcional y Genómica, Consejo Superior de Investigaciones Cinetificas/Universidad de Salamanca, Salamanca, Spain; 5 Laboratory of Electron Microscopy/Bio-imaging Centre, and Department of Chemical and Biological Sciences, Faculty of Sciences, Japan Women’s University, Mejirodai, Bunkyo-ku, Tokyo, Japan; 6 NPO Integrated Imaging Research Support, Hirakawa-cho, Chiyoda-ku, Tokyo, Japan; Howard Hughes Medical Institute and Vanderbilt University School of Medicine, UNITED STATES

## Abstract

Cytokinesis in many organisms requires a plasma membrane anchored actomyosin ring, whose contraction facilitates cell division. In yeast and fungi, actomyosin ring constriction is also coordinated with division septum assembly. How the actomyosin ring interacts with the plasma membrane and the plasma membrane-localized septum synthesizing machinery remains poorly understood. In *Schizosaccharomyces pombe*, an attractive model organism to study cytokinesis, the β-1,3-glucan synthase Cps1p / Bgs1p, an integral membrane protein, localizes to the plasma membrane overlying the actomyosin ring and is required for primary septum synthesis. Through a high-dosage suppressor screen we identified an essential gene, *sbg1*^*+*^ (**s**uppressor of **b**eta **g**lucan **s**ynthase 1), which suppressed the colony formation defect of Bgs1-defective *cps1-191* mutant at higher temperatures. Sbg1p, an integral membrane protein, localizes to the cell ends and to the division site. Sbg1p and Bgs1p physically interact and are dependent on each other to localize to the division site. Loss of Sbg1p results in an unstable actomyosin ring that unravels and slides, leading to an inability to deposit a single contiguous division septum and an important reduction of the β-1,3-glucan proportion in the cell wall, coincident with that observed in the *cps1-191* mutant. Sbg1p shows genetic and / or physical interaction with Rga7p, Imp2p, Cdc15p, and Pxl1p, proteins known to be required for actomyosin ring integrity and efficient septum synthesis. This study establishes Sbg1p as a key member of a group of proteins that link the plasma membrane, the actomyosin ring, and the division septum assembly machinery in fission yeast.

## Author Summary

Cell division in many organisms requires the function of an actomyosin ring, an apparatus that resembles the force generating machinery in the muscle. This ring apparatus is attached to the cell periphery (cell membranes) such that when it contracts, it brings the cell periphery together with it, leading to cell division. How the actomyosin ring is attached to the cell membrane at the division site is unknown. In this manuscript, we identify and describe Sbg1, a protein that links the actomyosin ring and the cell membranes since Sbg1 has a sequence that allows it to be inserted into the cell membrane. Sbg1 specifically localizes to the cell division site and also cooperates with a cell wall biosynthetic enzyme Bgs1 to achieve cell division. Consistently, in the absence of Sbg1, cells fail to divide leading to lethality. Sbg1 interacts with a number of cell division proteins, such as Cdc15, Rga7, Imp2, and Pxl1, to achieve its function as a bridge between the cell membrane and the actomyosin ring. Our work identifies a direct molecular link between the actomyosin ring and the cell membranes, explaining how ring contraction leads to inward movement of the cell periphery.

## Introduction

Cytokinesis is the terminal step in the cell cycle during which two cells are formed starting from one. Fungi and metazoans use a plasma membrane anchored actomyosin-based contractile ring to mark the cell division site and contraction of the actomyosin ring generates a part of the tension required to divide the cell [1–3]. Furthermore, in fungi, actomyosin ring contraction is coordinated with assembly of a carbohydrate rich cell wall / division septum outside of the plasma membrane that provides mechanical strength to the cells [4–8]. How the actomyosin ring is attached to the plasma membrane and how actomyosin ring contraction is coupled to division septum and cell wall synthesis are not fully understood.

Over the last two decades, the fission yeast *Schizosaccharomyces pombe* has emerged as an attractive model organism for the study of actomyosin ring dependent cell division and its coordination with division septum and cell wall assembly [9,10]. *S*. *pombe* grows by elongation at cell ends and divides by medial fission. The site of division is defined by inhibitory cues originating from the cell end and by stimulatory cues from the position of the interphase nucleus [11]. Cell division is brought about by a membrane anchored actomyosin ring whose contraction is tightly coupled to septum synthesis. Growing evidence in this organism suggests that while the actomyosin ring generates tension, the rate of growth of the septum cell wall determines the rate of contraction of the actomyosin ring [12,13]. In addition, some studies have proposed that the majority of tension for cytokinesis is generated by cell wall growth rather than from actomyosin ring contraction [14]. Furthermore, Muñoz et al. suggest that force required for cytokinesis is not provided either by ingression of the septum or by ring contraction but only by plasma membrane growth [8]. The mechanisms by which the actomyosin ring, plasma membrane, and septum wall synthesis are coordinated are a subject of major interest.

The division septum is a three-layered structure with a primary septum in the middle flanked by secondary septum on either side [15,16]. The primary and secondary septa are biochemically distinct allowing for specific action of α- and β-glucanases at cell separation [17–19]. The primary septum is composed chiefly of a special linear β-1,3-glucan almost exclusively detected only in this septum, along with branched β-1,3-glucan and α-1,3-glucan [20,21]. The protein Cps1p / Bgs1p, one of the four essential catalytic subunits of β-1,3-glucan synthase, is responsible for the synthesis of this linear β-1,3-glucan at the primary septum [22–27]. Branched β-1,3-glucan and α-1,3-glucan are present at both septa and the cell wall, while branched β-1,6-glucan is observed in the secondary septum and cell wall [21,28]. Bgs4p, the major β-1,3-glucan synthase, synthesizes branched β-1,3-glucan, which is essential for connecting the actomyosin ring to the cell wall and thus maintaining rigidity in the primary septum [8,28,29]. Ags1p incorporates α-glucan into the septum and the cell wall, providing stability and structural support to the primary septum during cell separation [28,30,31]. α-glucan is also required for correct secondary septum formation. Thus, various cell wall components play an essential role in providing rigidity to the division septum and maintaining cell integrity throughout the life of the cell.

Recent literature points towards the existence of a network of proteins that anchor the actomyosin ring to the membrane and link it to the septum synthesis machinery. Current work has identified the presence of Cdc15p, Imp2p, Pxl1p, Fic1p, Rga7p, Bgs1p, Bgs4p, Ags1p, among others in this network, with the possibility of even more proteins working together for this crucial step [8,28,32–39]. Cdc15p and Imp2p, both F-BAR proteins, cooperate in providing stability to the actomyosin ring via their SH3 domains [33,40]. Cdc15p has also been shown to transport Bgs1p to the cell middle [41]. Absence of the LIM-domain protein Pxl1p, leads to defective actomyosin rings that are irregular and unable to coalesce completely [34,42]. Absence of this protein also affects the anchorage of actomyosin ring at the cell middle, resulting in sliding of actomyosin rings and a high percentage of cells with off-centre septa [35]. Cells defective in the F-BAR protein Rga7p exhibit improperly disassembled rings and aberrant septa. The septum synthesis machinery also has a direct role in ring stabilization [8,28,35,41]. At the restrictive temperature, mutant *cps1-191* cells display very slow actomyosin ring constriction or a complete failure of constriction [24]. Concomitantly, a defective septum is synthesized, possibly because of lack of proper Bgs1p function or lack of the force generated from the ingressing septum [14,35]. This defect also results in less efficient anchoring of the actomyosin ring with a proportion of rings sliding towards one cell end in *cps1-191* cells [35,41]. Repression of Pxl1p expression in *cps1-191* background aggravates the ring sliding phenotype while the double mutant *cps1-191 cdc15SH3Δ* is synthetic lethal [35]. These results suggest collaboration between ring proteins and Bgs1p for efficient ring anchorage, ring integrity, and septation.

With the aim to identify other molecules involved in septum synthesis and / or contributing to the integrity of the actomyosin ring, we used a high-dosage suppressor screen to identify suppressors of the *cps1-191* mutant. Here we report one such suppressor, *SPBP22H7*.*03*, *sbg1*^*+*^ (suppressor of beta glucan synthase 1). Our characterization of Sbg1p reveals it to be an essential integral membrane protein, which is a crucial link between the actomyosin ring, plasma membrane and the primary septum synthesizing machinery.

## Results

### *sbg1*^*+*^ is a multi-copy suppressor of mutant *cps1-191*

**Fig 1:**
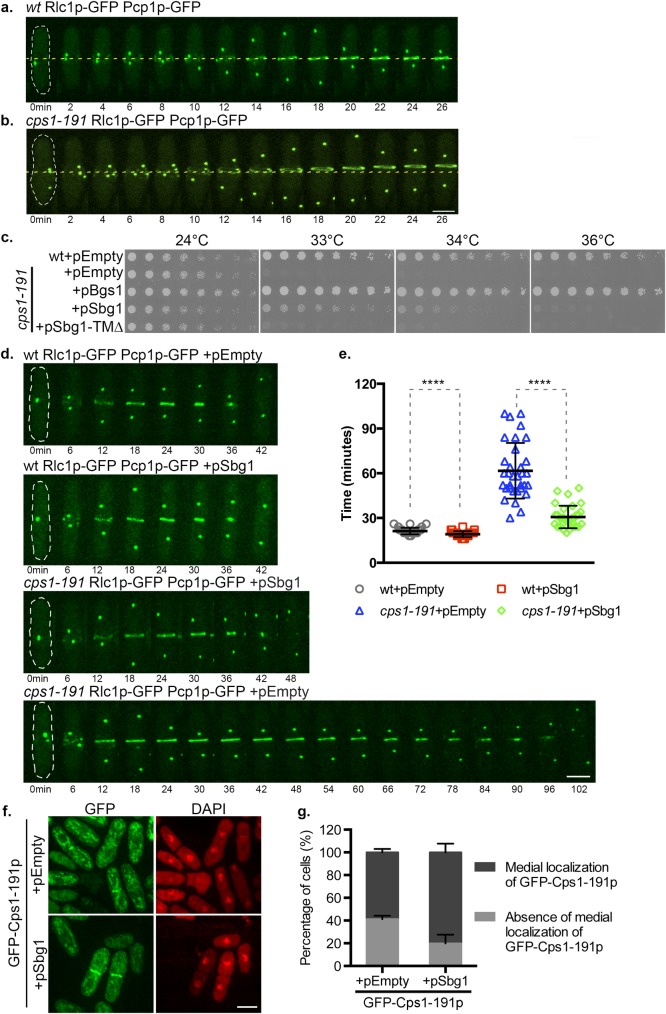
Multi-copy expression of *sbg1*^*+*^ rescues *cps1-191*. (A) Time-lapse maximum Z projection spinning disk confocal montage of an actomyosin ring in wt Rlc1p-GFP Pcp1p-GFP (MBY 5732) after 2 hr at 36°C. No ring sliding was observed (n = 2, 8cells). 0min indicates time of spindle pole body duplication. Green, Rlc1p-GFP Pcp1p-GFP. (B) Time-lapse maximum Z projection spinning disk confocal montage of a sliding actomyosin ring in *cps1-191* Rlc1p-GFP Pcp1p-GFP (MBY 5730) after 2 hr at 36°C. Average no of cells with sliding rings: 20.8%±5.9% (n = 2, ≥30cells). 0min indicates time of spindle pole body duplication. Green, Rlc1p-GFP Pcp1p-GFP. (C) Spot assay comparing the viability of the strains of indicated genotypes. Cultures of the strains: wt+pEmpty (MBY8558), *cps1-191+*pEmpty (MBY8944), *cps1-191+*pSbg1 (MBY8946) and *cps1-191+*pBgs1 *(*MBY8947) were grown overnight at 24°C, were serially diluted in two-fold steps and spotted on minimal media agar plates without leucine and incubated at various growth temperatures. (D) Time-lapse maximum Z projection spinning disk confocal montages of actomyosin ring in the indicated strains: wt Rlc1p-GFP Pcp1p-GFP+pEmpty (MBY9493), wt Rlc1p-GFP Pcp1p-GFP+pSbg1 (MBY9508), *cps1-191* Rlc1p-GFP Pcp1p-GFP+pSbg1 (MBY9456) and *cps1-191* Rlc1p-GFP Pcp1p-GFP+pEmpty (MBY9454) after 3.5 hr at 34°C. 0min indicates time of spindle pole body duplication. Green, Rlc1p-GFP Pcp1p-GFP. (E) Graph shows the time taken (in minutes) for the ring to constrict in the indicated strains in (d). p-value<0.0001 indicated by **** (two-tailed T-test). (F) Maximum Z projection spinning disk confocal images of indicated strains: GFP-Cps1-191p+pEmpty_his3 (MBY9188) and GFP-Cps1-191p+pSbg1_his3 (MBY9193) after 6 hr at 34°C. Cells were fixed and stained with DAPI to identify binucleate cells. (G) Quantification for the presence or absence of medial GFP-Cps1-191p localization at 34°C for experiment in (f). (n = 3, ≥340cells). Scale bar 5μm. Error bars indicate S.D.

*cps1-191* is defective in primary septum synthesis at the restrictive temperature and arrests as a binucleate cell with an unconstricted actomyosin ring [24]. As reported previously [35,41], we confirmed in *cps1-191* cells expressing Rlc1p-GFP (marker for actomyosin ring) and Pcp1p-GFP (spindle pole body marker indicating mitotic progression) that the actomyosin ring failed to constrict and eventually slid off the cell middle at 36°C (Fig 1B). Such a ring sliding phenotype was not observed in wild type cells imaged under similar conditions (Fig 1A).

We performed a plasmid based multi-copy suppressor screen to isolate genes that, upon overexpression, could suppress the growth defect of *cps1-191* at a semi-restrictive temperature of 34°C. We identified a gene (*SPBP22H7*.*03*) encoding a predicted single pass transmembrane protein, that restored the ability of *cps1-191* to form colonies at 34°C (Fig 1C). We named this gene *sbg1*^*+*^ (**s**uppressor of **b**eta **g**lucan synthase 1). However, such increased expression of *sbg1*^*+*^ could neither rescue *cps1-191* at 36°C nor could it restore viability of *bgs1Δ* cells, suggesting that Sbg1p is not a bypass suppressor of *bgs1* mutants. Multi-copy expression of a version of Sbg1p lacking the predicted transmembrane domain (amino acid residues 150–172; Sbg1-TMΔp) also did not suppress *cps1*-*191* defects at 34°C, suggesting that the potential membrane localization of Sbg1p is important for its function.

### Multi-copy expression of *sbg1*^*+*^ promotes better ring constriction and medial retention of Cps1-191p in mutant cells

Since high copy expression of *sbg1*^*+*^ rescued defects in colony formation, we first investigated if overproduction of Sbg1p corrected the following two defects in *cps1*-*191* at 34°C: impaired ring constriction and impaired localization of Cps1-191p (the product of *cps1-191*). Towards this goal, we first imaged kinetics of actomyosin ring constriction (using Rlc1p-GFP as a marker) in wild type and *cps1*-*191* cells harboring an empty vector (pAL-Empty) or pSbg1 (pAL-Sbg1) at the temperature of 34°C and also determined the time taken for ring constriction (defined as time from a completely assembled ring to complete constriction) (Fig 1D and 1E). Wild type cells with an empty vector required 21.23±2.19 min to constrict their actomyosin rings, while multi-copy expression of *sbg1*^*+*^ in wild type cells led to a mildly increased contraction rate (~9%). We also observed that *cps1*-*191* cells carrying an empty plasmid required 61.69±18.64 minutes for ring constriction under the semi-restrictive conditions. However, overproduction of Sbg1p improved ring constriction dynamics dramatically in *cps1-191* cells. These rescued cells completed constriction in 30.68±7.58 minutes—half the time taken by mutant cells and the constriction time was only slightly longer than in wild type cells. These results showed that excess Sbg1p strongly accelerates actomyosin ring constriction in *cps1-191* mutant.

Previous work has shown that the protein product of *cps1*-*191* fails to localize properly to the division site even at permissive temperature (25°C), though actomyosin ring assembly is normal in these cells [35]. We addressed if overexpression of Sbg1p restored localization of Cps1-191p to the division site. To test this possibility, strains expressing GFP-Cps1-191p and carrying an empty plasmid or pSbg1 were imaged to determine the localization of mutant GFP-Cps1-191p at the semi-restrictive temperature (Fig 1F and 1G). At the semi-restrictive temperature, 59% of binucleate mutant cells displayed medial localization of GFP-Cps1-191p. However, in the rescued *cps1-191* cells carrying pSbg1, this number increased to ~80% (Fig 1F and 1G). We conclude that an excess of Sbg1p facilitates better retention of Cps1-191p at the cell division site.

### Multi-copy expression of *sbg1*^*+*^ corrects septum synthesis defect of *cps1-191*

**Fig 2:**
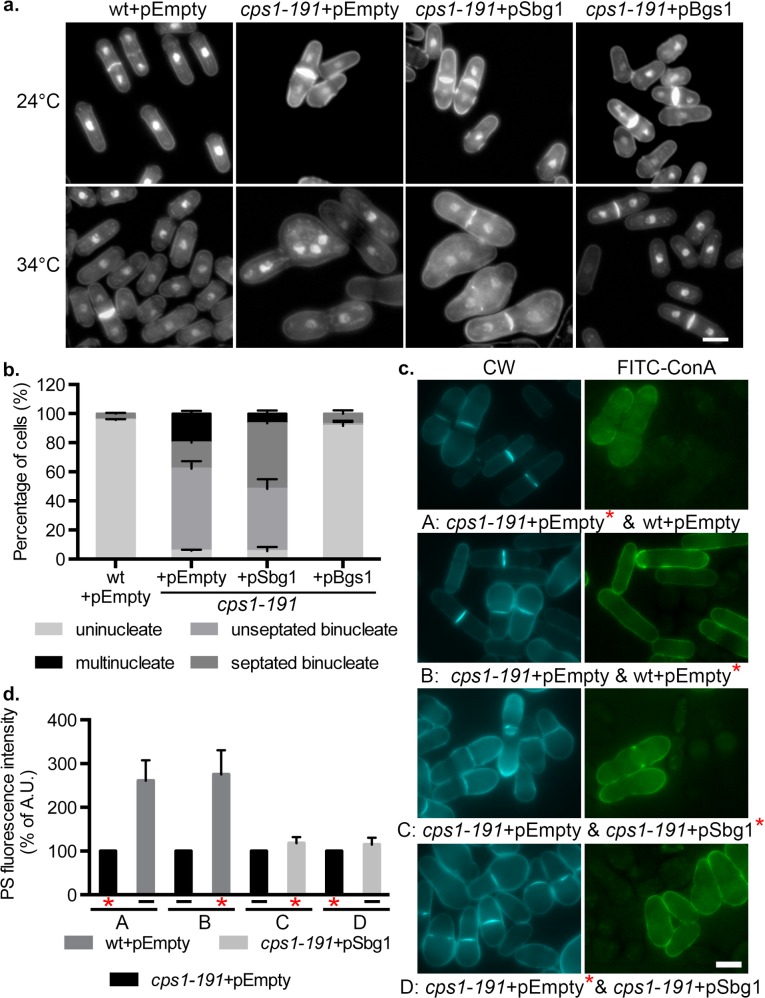
Multi-copy expression of *sbg1* corrects septum synthesis defect of *cps1-191*. (A) Aniline blue and DAPI images of medial plane of cells of indicated genotype wt+pEmpty (MBY8558), *cps1-191+*pEmpty (MBY8944), *cps1-191+*pSbg1 (MBY8946) and *cps1-191+*pBgs1 *(*MBY8947), fixed at 24°C or after shift to 34°C for 16 hr. (B) Quantification of 34°C phenotypes in (a). *cps1-191*+pEmpty vs *cps1-191*+pSbg1 septated binucleate cells: p-value = 0.001 (two-tailed T-test). (n = 3, ≥200cells). (C) Calcofluor white (CW) images of the indicated strains: wt+pEmpty (MBY8558), *cps1-191+*pEmpty (MBY8944) and *cps1-191+*pSbg1 (MBY8946) after 16 hr at 34°C, with one strain also stained with Concanavalin A (ConA) conjugated with FITC (labelled strain indicated with an asterisk*). A-D refer to the different combinations analyzed and are stated in the figure. (D) Quantification of observed Calcofluor white fluorescence intensity for experiment done in (c). Percentage of PS fluorescence intensity normalized to septum length of each strain compared to that of *cps1-191*+pEmpty strain, which was assigned a value of 100%. (n≥24cells). Scale bar 5μm. Error bars indicate S.D.

The main defect observed in *cps1-191* mutant is the inability to synthesize the primary septum. Cells were stained with aniline blue and DAPI to analyze primary septum structure and nuclei, respectively, to determine whether multi-copy expression of *sbg1*^***+***^ restored septum synthesis in the *cps1-191* mutant. Cells were shifted to the temperature of 34°C for 16 hr in selective minimal medium. At this temperature, wild type cells carrying an empty plasmid maintained normal cylindrical morphology, and assembled a centrally placed division septum (Fig 2A and 2B). As expected, *cps1*-*191* cells carrying an empty plasmid accumulated 56% unseptated binucleate cells and around 20% cells with multiple nuclei. Rescued *cps1-191* cells bearing pSbg1 plasmid showed 43% unseptated binucleate cells and only 7% multinucleate cells. Interestingly, consistent with the ability of *cps1*-*191* cells with pSbg1 to form colonies, these cells displayed a dramatic increase in the number of septated binucleate cells (45%) as compared to only 18% in *cps1*-*191* cells with an empty plasmid. These data suggested that overproduction of Sbg1p led to an increase in the proportion of *cps1*-*191* cells assembling a division septum.

### Multi-copy expression of *sbg1*^*+*^ improves the quality of the primary septum synthesized in *cps1-191* cells

In wild type cells, Bgs1p is responsible for the synthesis of the linear β-1,3-glucan at the primary septum. In cells defective in Bgs1p, the division site shows a thick secondary septum that may compensate for the lack or partial defects of primary septum [20]. An even thicker secondary septum is observed on complete absence of Bgs1p to compensate for the complete lack of primary septum in this scenario [20]. As multi-copy expression of *sbg1*^*+*^ in *cps1-191* led to an increase in the number of septated cells, we wanted to determine if it also facilitated better incorporation of linear β-1,3-glucan into the division septum. To this end, we used the fluorophore calcofluor white (CW) which specifically binds to linear β-1,3-glucan at the primary septum and measured its fluorescence intensity at the septum as a tool to assess the amounts of linear glucan [20,43]. Cells were shifted to 34°C for 16 hr, and two different strains were mixed together, one of them being labeled with FITC-concanavalin A (binds to outer galactomannan layer) to differentiate between them (Fig 2C). Fluorescence intensity was calculated as arbitrary units using ImageJ and the final value was determined as intensity normalized to the length of the septum. Wild type cells carrying empty plasmid and *cps1-191* cells with pSbg1 were both compared to mutant *cps1-191* cells with empty plasmid, whose intensity was set to 100% and used as a reference in all combinations of analyses (Fig 2D). We observed fluorescence intensity to be much higher in wild type cells at 270% relative to that of mutant *cps1-191* cells. In the opposite analysis, the primary septum of *cps1-191* cells showed only 39% intensity relative to wild type primary septum intensity. Interestingly, fluorescence intensity of CW in rescued cells was also slightly higher at around 118% relative to that of mutant cells (and 46% of wild type intensity). The percentage of CW-stained complete septa also increased from 43% in *cps1-191* cells to 67% in rescued cells. This analysis implied that upon multi-copy expression of *sbg1*^*+*^, *cps1-191* could synthesize a primary septum that was more enriched in linear β-1,3-glucan, the product of Bgs1p, thus facilitating septum progression and completion.

*S*. *pombe* divides by fission at the cell middle resulting in the production of two equal sized daughter cells. We determined if multi-copy expression of *sbg1*^*+*^ in *cps1-191* cells restored the medial placement of division septum. To this end, we compared displacement of septa from the cell middle in *cps1-191* cells carrying pSbg1 to that in wild type cells harboring an empty plasmid at 34°C after 16 hr (S1A Fig). The *cps1-191* mutant lacks proper septum synthesis and was excluded from the analysis. Wild type *S*. *pombe* cells deposited a medially placed septum (0–0.5μm displacement range) at this temperature in approximately 91% of the cells. Overall, about 58% of the septa in *cps1*-*191* with pSbg1 were in the 0–0.5μm range, while 31% fell in the 0.5–1 μm range, and remainder displaced even further from the cell center. Through this data we concluded that multi-copy expression of *sbg1*^*+*^ enabled proper synthesis of a medial septum in a large proportion of *cps1-191* cells.

### Multi-copy expression of *sbg1*^*+*^ improves cell wall structures in the rescued cells

We considered the possibility that *cps1-191* mutant may show aniline blue staining but the septum structure may be incomplete or aberrant in morphology. To this end, we analyzed 3D reconstruction confocal images of cells stained with aniline blue (S1B Fig). The aniline blue stained structures were found to be one of the following- a complete flat disc, a complete curved disc or a complete wavy irregular disc. Septa that were in the process of being synthesized were observed to be either regular in a clear circular shape or very irregular. At 34°C, control wild type cells showed complete flat (24%), complete curved (29%) or an open regular (46%) septum. On the other hand, *cps1-191* cells with empty plasmid displayed either open irregular (75%) or complete wavy (25%) septum. Upon multi-copy expression of *sbg1*^*+*^ in *cps1-191* cells, around 37% of the cells now showed either complete septum with or without a curvature or an open regular septum. Among the rest, 33% of the cells were able to synthesize a complete wavy septum while only 31% cells displayed irregular open septum. These results further established that multi-copy expression of *sbg1*^*+*^ enabled completion of septum synthesis while improving the quality of the septum.

**Fig 3:**
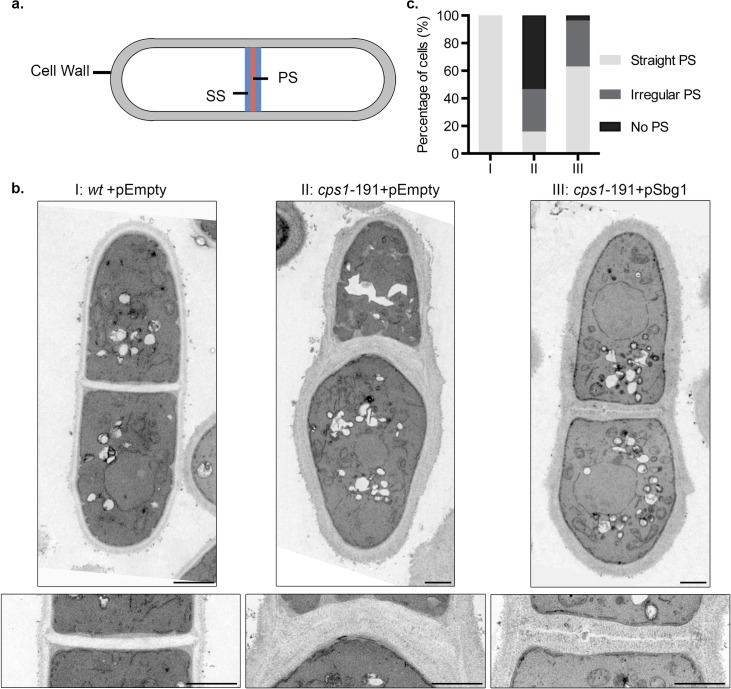
Multi-copy expression of *sbg1*^*+*^ improves cell wall structures of *cps1-191*. (A) Illustration depicting a longitudinal section of a septated wild type cell with both primary septum (PS) and secondary septum (SS). (B) Transmission electron microscopy images of septated cells of the indicated strains, I: wt+pEmpty (MBY8558), II: *cps1-191+*pEmpty (MBY8944) and III: *cps1-191+*pSbg1 (MBY8946) after 16 hr at 34°C. The bottom panel displays the division septum at a higher magnification. (C) Quantification of septated cells observed from the transmission electron microscopy images. (n≥14cells). Scale bar 1μm.

To get a more detailed understanding of the septum structure, we sought to determine the ultrastructure of septum and cell wall in rescued *cps1-191* cells carrying pSbg1 by transmission electron microscopy (Fig 3B and 3C). We analysed cells that were longitudinally cut and displayed a completed division septum. We specifically examined the presence or absence of an electron-transparent primary septum component of the division septum. If a primary septum was present, we determined if it was straight and rigid as in wild type cells or had defects and showed irregularities in its structure. Wild type cells carrying the empty plasmid displayed a clear three-layered division septum structure, with an electron-transparent primary septum in the middle flanked by electron-dense secondary septum on either side (Fig 3A, 3Bi and 3C). Consistent with previous results, about 50% of the *cps1-191* septated cells lacked a primary septum but displayed thick deposition of the secondary septa, whereas 30% of septated cells showed an irregular primary septum (Fig 3Bii and 3C) while the rest showed a normal straight primary septum. The normal straight primary septa may have been synthesized before the shift up or resulted from residual Cps1-191p activity at 34°C. However, rescued septated *cps1-191* cells carrying pSbg1 showed a correction in these septum defects (Fig 3Biii and 3C). Around 60% of septated cells were able to synthesize a straight primary septum, 30% of septated cells showed an irregular primary septum and only 5% of septated cells did not have any primary septum. In conclusion, multi-copy expression of *sbg1*^*+*^ enabled synthesis of primary septum in the mutant *cps1-191*.

Changes in cell wall / division septum ultrastructures are a direct indication of perturbations in cell wall composition. We investigated if multi-copy expression of *sbg1*^*+*^ modified the cell wall composition of *cps1-191* cells resulting in improved division septum assembly therein. Towards this goal, we determined the cell wall composition of mutant and rescued cells (S1 Table) [8,44]. At 34°C, *cps1-191* cells with an empty plasmid demonstrated a significant increase (144% compared to the wild type cell wall amount at 34°C–compare 36.4% of the cell wall amount in the mutant vs 25.7% of cell wall amount in wild type cells, S1 Table) in the cell wall in general and also an increase in α-1,3-glucan and β-1,6-glucan levels in the cell wall as compared to wild type cells (α-1,3-glucan: 38.3% vs 24.8% of the cell wall, and β-1,6-glucan: 6.1% vs 2.9% of the cell wall respectively, S1 Table). In yeast, an increase in other cell wall components, usually α-1,3-glucan, as a compensatory mechanism is typically observed in cell wall mutants affected in different types of polysaccharides [8,20,28,45,46]. We also observed that mutant *cps1-191* cells displayed a much lower amount of β-1,3-glucan even at 24°C (87% of wild type, 47.8% vs 55.0% of the cell wall at 24°C, S1 Table). The amount of β-1,3-glucan was further reduced at 34°C (81% of wild type, 48.9% vs 59.8% of the cell wall at 34°C, S1 Table). This reduction of β-1,3-glucan corresponds to combined branched and linear β-1,3-glucan from Bgs1p, Bgs4p and possibly Bgs3p. The total β-1,3-glucan amount in the cell wall showed a slight and statistically-significant increase upon multi-copy expression of *sbg1*^*+*^ in *cps1-191* cells at 34°C (S1 Table). The increase in β-1,3-glucan levels are not striking since the Bgs1p-promoted primary septum linear β-1,3-glucan makes up only a small fraction of the total β-1,3-glucan in the cell wall. Nonetheless, this analysis further reinforced our notion that multi-copy expression of *sbg1*^*+*^ facilitated better septum synthesis by increasing primary septum linear β-1,3-glucan synthesis (also see Fig 2C and 2D) and concomitantly decreasing the compensatory alterations of other septum and cell wall polymers (α-1,3- and β-1,6-glucans).

### Sbg1p, an integral membrane protein, physically interacts with Bgs1p

Sbg1p possesses a transmembrane domain and is predicted to be an integral membrane protein. To test the association of Sbg1p with the membrane, we used a strain in which *hyg*^*r*^-GFP was fused at the N-terminus of Sbg1p by marker fusion tagging [47]. The strain also expressed HA-Bgs1p, a known integral membrane protein with 14 to 16 transmembrane domains [22,26]. Cells were treated in 8 different ways to investigate the association of Sbg1p with the membrane [48]. Cell proteins were extracted with lysis buffer alone, or buffer supplemented with urea, NaCl, Na_2_CO_3_, Tween 20, digitonin, TritonX-100, or SDS (S2A Fig). Following ultracentrifugation, the pellet and supernatant fractions were resolved on a SDS-PAGE gel and the lower part of the membrane was probed for Sbg1p (with antibodies against GFP) and the upper part for Bgs1p (with antibodies against HA). Bgs1p was only solubilized upon treatment with SDS confirming it to be an integral membrane protein, as previously shown by Liu et al. [26]. *hyg*^*r*^ -GFP-Sbg1p, despite its predicted molecular weight of 85kDa, was detected as a ~ 70 kDa band and was solubilized fully only upon SDS treatment, establishing it to be an integral membrane protein. The untagged wild type cells did not show any bands corresponding to either of the proteins. These experiments established that Sbg1p, like Bgs1p, was an integral membrane protein.

**Fig 4:**
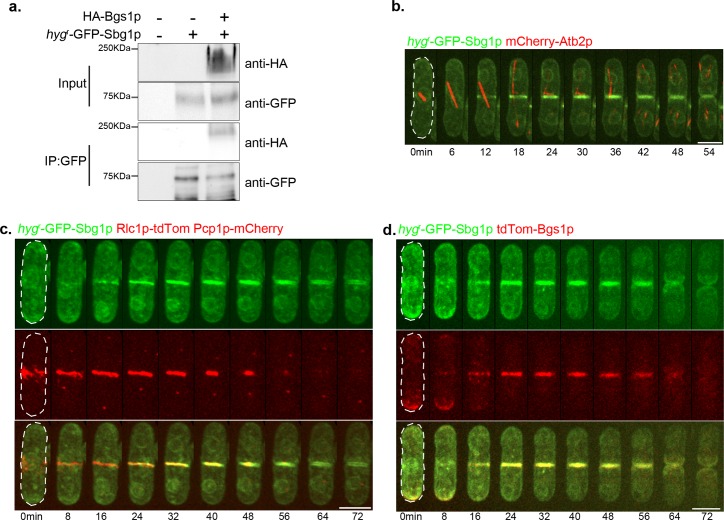
Characterization of Sbg1p. (A) Bgs1p physically interacts with Sbg1p. Solubilized membrane proteins from the indicated strains: wt (MBY192), *hyg*^*r*^-GFP-Sbg1p (MBY8967) and HA-Bgs1p *hyg*^*r*^-GFP-Sbg1p (MBY9241) were immunoprecipitated (IP) with anti-GFP antibodies. Solubilized membrane proteins (input, top) and IP (bottom) were transferred to the same membrane and blotted with monoclonal anti-HA antibodies. (B) Time-lapse maximum Z projection spinning disk confocal montage of the indicated strain *hyg*^*r*^-GFP-Sbg1p mCherry-Atb2p (MBY8977). Green, *hyg*^*r*^-GFP-Sbg1p. Red, mCherry-Atb2p. 0min marks spindle pole body duplication as judged by a short microtubule spindle. (C) Time-lapse maximum Z projection spinning disk confocal montage of the indicated strain *hyg*^*r*^-GFP-Sbg1p Rlc1p-tdTomato Pcp1p-mCherry (MBY9389). Green, *hyg*^*r*^-GFP-Sbg1p. Red, Rlc1p-tdTomato Pcp1p-mCherry. 0min marks spindle pole body duplication as judged by Pcp1p signal. (D) Time-lapse maximum Z projection spinning disk confocal montage of the indicated strain *hyg*^*r*^-GFP-Sbg1p tdTomato-Bgs1p (MBY9006). Green, *hyg*^*r*^-GFP-Sbg1p. Red, tdTomato-Bgs1p. 0min marks initiation of nuclear division as judged by Sbg1p signal at nuclear periphery. Scale bar 5μm.

Given that Sbg1p was an integral membrane protein and the genetic interaction of *sbg1* with *bgs1*, we tested if Bgs1p and Sbg1p interacted physically. Previous work has shown that Bgs1p physically interacts with Ags1p, which encodes a α-1,3-glucan synthase [28]. We found that immune complexes generated with antibodies against GFP, from cells expressing *hyg*^*r*^-GFP-Sbg1p and HA-Bgs1p or cells expressing GFP-Ags1p and HA-Bgs1p, both contained HA-Bgs1p (Fig 4A and S2B Fig). HA-Bgs1p was not recovered in immune complexes generated with GFP antibodies in strains that only expressed HA-Bgs1p. Furthermore, bands of size similar to HA-Bgs1p were not found in wild type cells in which Bgs1p was not tagged. These experiments established that Bgs1p likely physically interacted with Sbg1p.

### Sbg1p, an essential protein, localizes to the division septum membrane

The database for *S*. *pombe*, PomBase, suggests that *sbg1*^*+*^ is an essential gene as a genome-wide deletion collection describes *sbg1Δ* spores incapable of germination [49–51]. Therefore, heterozygous diploids were obtained in which one copy of *sbg1* (between sequences encoding amino-acids 28–173) was replaced with the geneticin resistance marker. These diploids were sporulated and subjected to tetrad analysis and indeed a 2:2 segregation of growth was observed on rich medium (S2C Fig). A 2:2 segregation of growth was also observed on plates supplemented with 1.2M sorbitol, a known osmotic stabilizer that is able to partially suppress cell wall defects. Microscopic analysis revealed that dying presumed *sbg1Δ* cells had a rounded and swollen morphology and accumulated multiple nuclei, similar to *bgs1Δ* cells [20,24]. *sbg1Δ* cells failed to synthesize a septum but progressed through several mitotic cycles, with 30% cells accumulating more than 4 nuclei and 3% cells lysing at 24 hr. This indicated that Sbg1p is essential for septum synthesis in fission yeast.

We next wanted to determine the localization of Sbg1p in wild type cells. To this end we imaged cells expressing *hyg*^*r*^-GFP-Sbg1p and mCherry-Atb2p (Fig 4B). We used tubulin as a cell cycle stage marker to determine the localization of Sbg1p at different cell cycle stages. We observed enrichment of Sbg1p at the old end immediately after cell division. This was followed by Sbg1p appearing at both the cell ends. During and following nuclear division, Sbg1p was enriched at the medial cortex. Sbg1p localized as a ring initially and gradually ingressed as a faint disc and was more intense at the septum edge overlapping the ring as the cell underwent septation (Fig 4B). Sbg1p was also detected in the rim of the nucleus and fibrous structures in the cell, likely reflecting its localization to the endoplasmic reticulum en route its destination in the plasma membrane. Sbg1p localization appeared similar to that described previously for Bgs1p [27], which is consistent with the physical interaction between Sbg1p and Bgs1p.

We then determined the localization of Sbg1p relative to the actomyosin ring. We used Rlc1p-tdTomato as a marker for the ring with Pcp1p-mCherry at the spindle pole body to mark mitotic progression (Fig 4C). We observed Sbg1p signal to be overlapping with that of Rlc1p indicating it to be in the vicinity of the ring probably as a part of the septum synthesis machinery. To determine if indeed Sbg1p localized at the septum membrane and to check its localization relative to Bgs1p, we imaged cells expressing *hyg*^*r*^-GFP-Sbg1p and tdTomato-Bgs1p (Fig 4D). Localization of both Sbg1p and Bgs1p was strongly correlated and showed significant overlap, with both the proteins being enriched at the same poles and appearing at the cell middle during cytokinesis. Both the proteins localized at the septum membrane during cytokinesis.

### Temperature sensitive mutant *sbg1-3* deposits aberrant septum

**Fig 5:**
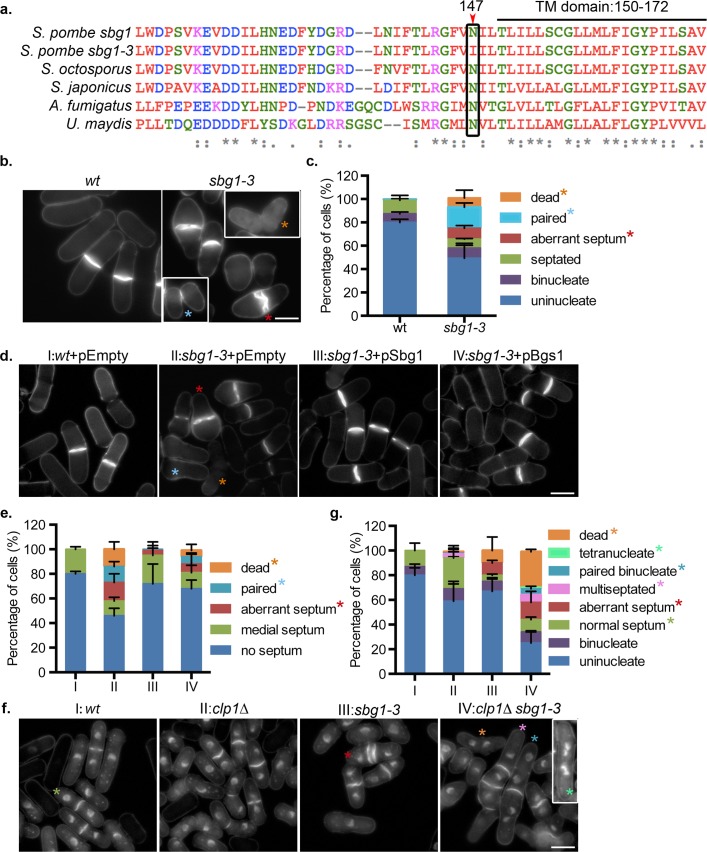
Temperature sensitive mutant *sbg1-3* deposits aberrant septa. (A) Multiple sequence alignment of *sbg1*, *sbg1-3* and related proteins from other filamentous fungi. Mutation in *sbg1-3* was identified at N147 as indicated in the image, just before the TM domain (residues 150–172). (B) Calcofluor white (CW) images of the medial plane of fixed cells from the indicated strains: wt (MBY192) and *sbg1-3* (MBY9359) after 6 hr at 36°C. Insets from other fields to show paired and dead cells. Coloured asterisks mark different phenotypes. (C) Quantification for experiment described in (b). (n = 3, ≥508 cells). (D) Calcofluor white (CW) images of the medial plane of fixed cells from the indicated strains: wt+pEmpty (MBY8558), *sbg1-3*+pEmpty (MBY9372), *sbg1-3*+pSbg1 (MBY9370) and *sbg1-3+*pBgs1 (MBY9366). Cells were grown overnight at 24°C in minimal media lacking leucine, shifted to YES for 2 hr and then shifted to 36°C for 6 hr. Coloured asterisks mark different phenotypes. (E) Quantification of experiment described in (d). (n = 2, ≥507 cells). (F) Aniline blue and DAPI images of medial plane of cells from the indicated strains: wt (MBY192), *imp2Δ* (MBY977), *sbg1-3* (MBY9359) and *imp2Δ sbg1-3* (MBY9358), fixed after shift to 36°C for 6 hr. Insets from another field to show tetranucleate cells. Coloured asterisks mark different phenotypes. (G) Quantification for experiment described in (f). (n = 3, ≥500 cells). Scale bar 5μm. Error bars indicate S.D.

We wanted to determine the role played by Sbg1p in the vegetative cell cycle. Given that spores lacking *sbg1* were inviable, we generated a temperature sensitive *sbg1* mutant using marker reconstitution mutagenesis [52]. A mutant, *sbg1-3*, was isolated and sequencing of the ORF identified a single point mutation, just before the predicted transmembrane domain, resulting in a change from a conserved asparagine residue to isoleucine at residue 147 (Fig 5A). To better characterize the mutant phenotype, cells were cultured in rich medium and analyzed by aniline blue staining after 6 hr at 36°C (Fig 5B). About 8% of the *sbg1-3* cells were dead and ~10% of the cells displayed an aberrant septum, sometimes showing a double septum structure (Fig 5C). About 18% of the *sbg1-3* cells showed cell separation defects and remained paired together, indicating an improperly formed septum that failed to be resolved. None of these defects were observed in wild type cells at the same temperature.

Given that multi-copy expression of *sbg1*^*+*^ rescued *cps1-191* mutant cells and in light of the physical interactions between Sbg1p and Bgs1p, we addressed if high dosage expression of *bgs1*^*+*^ could rescue *sbg1-3* (Fig 5D and 5E). At the restrictive temperature, *sbg1-3* cells with empty plasmid showed aberrant septa and paired cells. As expected, the mutant was completely rescued upon multi-copy expression of *sbg1*^*+*^. However, a high but incomplete rescue was observed upon multi-copy expression of *bgs1*^*+*^, with a small proportion of cells still displaying defects in the septum (Fig 5D and 5E). These results suggested that Sbg1p has a direct role in assembly of a normal primary septum and that Bgs1p and Sbg1p strongly collaborate in this process.

To confirm and study this strong interaction between Bgs1p and Sbg1p in the cell wall and septum synthesis, the cell wall defects caused by the *cps1-191* and *sbg1-3* mutations were examined at the high restrictive temperature of 36°C by cell wall fractionation analysis (S2 Table). The *cps1-191* mutation is strong and cell lysis appears after 5 hr at 36°C, and therefore the cell wall was analyzed after 4.5 hr at 36°C, immediately before lysis emergence. However, the *sbg1-3* mutation is mild and therefore the cell wall defects were examined after 24 hr at 36°C. Interestingly, both protein defects showed similar cell wall alterations except in the increase of cell wall amount which would be expected from the longer incubation time for *sbg1-3* (47% in *sbg1-3* and 36% in *cps1-191*). However, the defect in both proteins caused a similar decrease in β-1,3-glucan (72% in *cps1-191* and 75% in *sbg1-3*) and a similar compensatory increase in α-1,3-glucan (172% and 156%, respectively) and β-1,6-glucan (216% and 233%) and a decrease in galactomannan (51% and 55%) (S2 Table). This strong coincidence in cell wall β-1,3-glucan reduction and in each compensatory alteration, in both the affected polysaccharide and the altered proportion, suggests a close collaboration between Sbg1p and the primary septum linear β-1,3-glucan synthesis machinery.

The relatively weak phenotype of *sbg1-3* point mutation compared to *sbg1Δ* led us to investigate if the cytokinesis checkpoint was responsible for part of the restoration of viability in *sbg1-3*. Clp1p plays an important role in ensuring proper cytokinesis in cells with minor perturbation in the cytokinetic machinery, and deletion of Clp1p in such a background results in multinucleate cells and lethality [53,54]. Thus, we analyzed the double mutant *sbg1-3 clp1Δ* and interestingly observed that 12% cells were now either multinucleated or multiseptated, whereas neither of the single mutants showed such a phenotype (Fig 5F and 5G). The double mutant *sbg1-3 clp1Δ* also resulted in a higher percentage of cell death (29%) as compared to 10% in *sbg1-3* and none in *clp1Δ*. This suggested that the cytokinesis checkpoint may provide a means to increase the viability of *sbg1-3* cells.

### Sbg1p and Bgs1p depend on each other to localize to the division site and interact genetically

**Fig 6:**
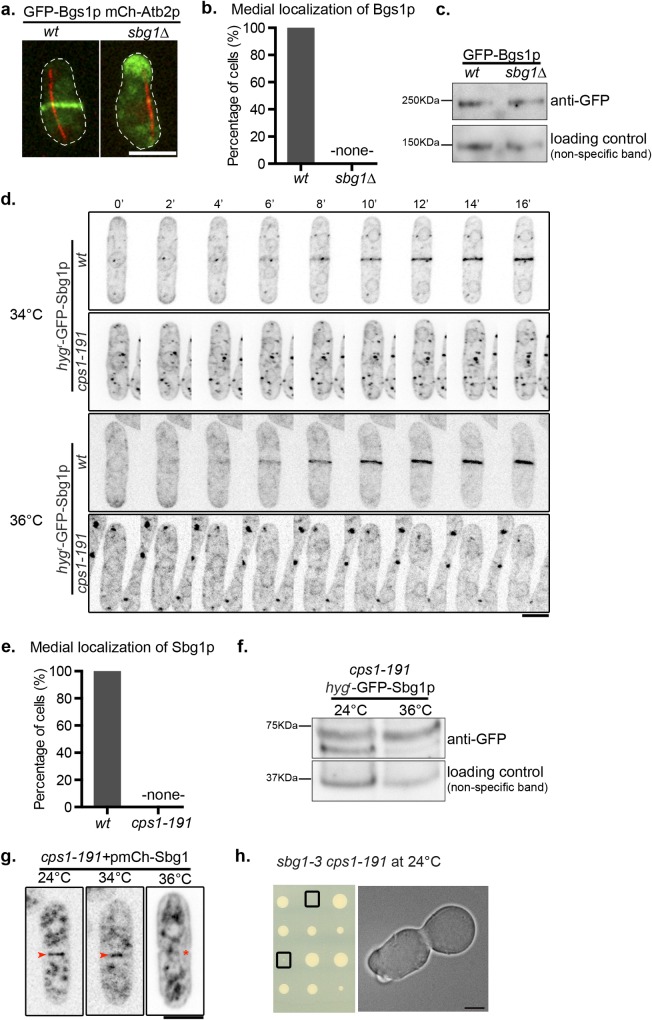
Localization dependencies between Sbg1p and Bgs1p. (A) Maximum Z projection spinning disk confocal images of germinated cells of the indicated genotype from diploid *sbg1Δ/sbg1*^*+*^ mCherry-Atb2p GFP-Bgs1p (MBY9160). Green, GFP-Bgs1p. Red, mCherry-Atb2p. (B) Quantification for the experiment described in (a). (C) Determination of Bgs1p protein levels in wild type cells (GFP-Bgs1p MBY8865) and sporulated *sbg1Δ*cells from diploid *sbg1Δ/sbg1*^*+*^ GFP-Bgs1p (MBY11106) respectively. (D) Time-lapse maximum Z projection spinning disk confocal montage of the indicated strains: *hyg*^*r*^-GFP-Sbg1p (MBY8967) and *cps1-191 hyg*^*r*^-GFP-Sbg1p (MBY9097) after 3 hr at 34°C and 36°C. Images are inverted for Sbg1p fluorescence. (E) Quantification for the experiment described in (d). (F) Determination of Sbg1p protein levels in *cps1-191 hyg*^*r*^-GFP-Sbg1p (MBY9097) cells at 24°C and 36°C after 4 hr. (G) Maximum Z projection spinning disk confocal images of *cps1-191* cells carrying multi-copy expression plasmid with mCherry at N-terminus of Sbg1p (*cps1-191*+pmCherry-Sbg1—MBY9285) at 24°C, 34°C and 36°C–shift up for 3 hr. Images are inverted for Sbg1p fluorescence. Arrowheads indicate presence of mCherry-Sbg1p signal at the division site while asterisk marks the absence of it. (n = 2, 50 cells). (H) Tetrad dissection analysis of a cross between *sbg1-3* and *cps1-191*. Boxes indicate double mutant *sbg1-3 cps1-191*. Scale bar 5μm.

Our findings indicated that Sbg1p and Bgs1p collaborate in efficient septum synthesis. We considered the possibility that these two proteins might function together in a single β-1,3-glucan synthase complex and that they might influence each other’s localization. Thus, we sought to determine if localization of these proteins was dependent on each other. Spores from *sbg1Δ/sbg1*^*+*^ diploid cells expressing GFP-Bgs1p and mCherry-Atb2p were inoculated in YES and YES with G418 (to select for *sbg1Δ*), in parallel (Fig 6A and 6B). We observed that cells in late anaphase (marked by a long spindle) in the YES grown culture displayed either medial localization of Bgs1p (putative germinating wild type spores) or mislocalized to either one cell end or as punctate structures throughout the cell (putative germinating *sbg1Δ* spores). However, all late mitotic germinating spores in YES+G418 displayed mislocalized Bgs1p and Bgs1p was never detected at the putative division site. From this analysis we were able to conclude that unlike wild type germinating spores that were able to localize Bgs1p to cell middle, *sbg1Δ* failed to do so. The absence of Bgs1p at the division site could in part be responsible for the inability of *sbg1Δ* to deposit a division septum.

We speculated if the absence of medial localization of Bgs1p in *sbg1Δ* mutant background could be a result of a decrease in Bgs1p protein expression levels. Heterozygous diploid *sbg1Δ/sbg1*^*+*^ expressing GFP-Bgs1p was sporulated and GFP-Bgs1p levels in germinating spores compared to GFP-Bgs1p in wild-type cells (Fig 6C). We did not observe a difference in the protein levels in these two cases, which is consistent with presence of Bgs1p fluorescent signal in *sbg1Δ* cells (Fig 6A).

Next, we assessed if localization of Sbg1p was affected in *cps1-191* mutant. We determined the localization of Sbg1p at 34°C (temperature at which overproduction of Sbg1p could rescue *cps1-191*) and at the full restrictive temperature of 36°C (Fig 6D and 6E). At both the temperatures, wild type cells displayed medial localization of Sbg1p after nuclear division (detected by nuclear Sbg1p localization). However, in mutant *cps1*-*191* cells, Sbg1p failed to localize to the cell middle after nuclear division at both the temperatures. Sbg1p localized to a number of punctate structures in addition to being present at the nuclear envelope / ER in *cps1*-*191* cells at 34°C. The function of these punctae or their relevance is not known. We also determined if protein levels of Sbg1p were affected in *cps1*-*191* cells (Fig 6F). We observed no difference in Sbg1p protein levels in *cps1-191* cells grown at 24°C or 36°C. Therefore, the failure to detect Sbg1p at the division site in *cps1*-*191* is not due to poor stability of Sbg1p at 34°C in *cps1*-*191*. We concluded that functional Bgs1p is required for medial localization of Sbg1p. This demonstrated a mutual dependency between Sbg1p and Bgs1p for proper medial localization and function in septum synthesis.

It was interesting to note that Sbg1p at endogenous levels did not localize to the division site in the *cps1-191* mutant background even at 34°C, but upon overexpression rescued *cps1-191* mutant, restoring primary septum synthesis and medial localization of Cps1-191p. We speculated that overexpression of Sbg1p may promote its own localization / maintenance at the division site in *cps1*-*191* at 34°C. We introduced a mCherry tag at the N-terminus of Sbg1p, which was expressed from a multi-copy expression plasmid and this plasmid was introduced into *cps1-191* cells (Fig 6G). At 24°C, we observed 91%±1.4% binucleate *cps1-191* cells (judged by localization of Sbg1p at the nuclear envelope) to show localization of overproduced Sbg1p at the division site. Remarkably, after shift up to 34°C for 3 hr, we observed 55%±4.2% binucleate *cps1-191* cells to show medial localization of Sbg1p. However, at 36°C, Sbg1p failed to localize to the cell middle even upon being overproduced with only 6%±2.8% binucleate *cps1-191* cells displaying medial localization of Sbg1p. We propose that it is this ability of overproduced Sbg1p to localize to the division site at 34°C that also drives the localization of mutant Cps1-191p protein to the division site (Fig 1F).

In light of the localization dependencies between Sbg1p and Bgs1p, we investigated if *sbg1*-3 and *cps1*-*191* showed genetic interactions. The meiotic progeny of *sbg1-3* and *cps1-191* were analyzed by tetrad analysis. In agreement with all data supporting interaction between Sbg1p and Bgs1p, the *cps1-191 sbg1-3* double mutant displayed very severe synthetic growth defects at permissive temperature of 24°C, resembling the phenotype of *bgs1Δ* and *sbg1Δ* cells (Fig 6H). All these data indicate a functional association and cooperation between Bgs1p and Sbg1p, which is necessary for proper cytokinesis in fission yeast.

### Actomyosin ring dynamics in *sbg1* mutants

**Fig 7:**
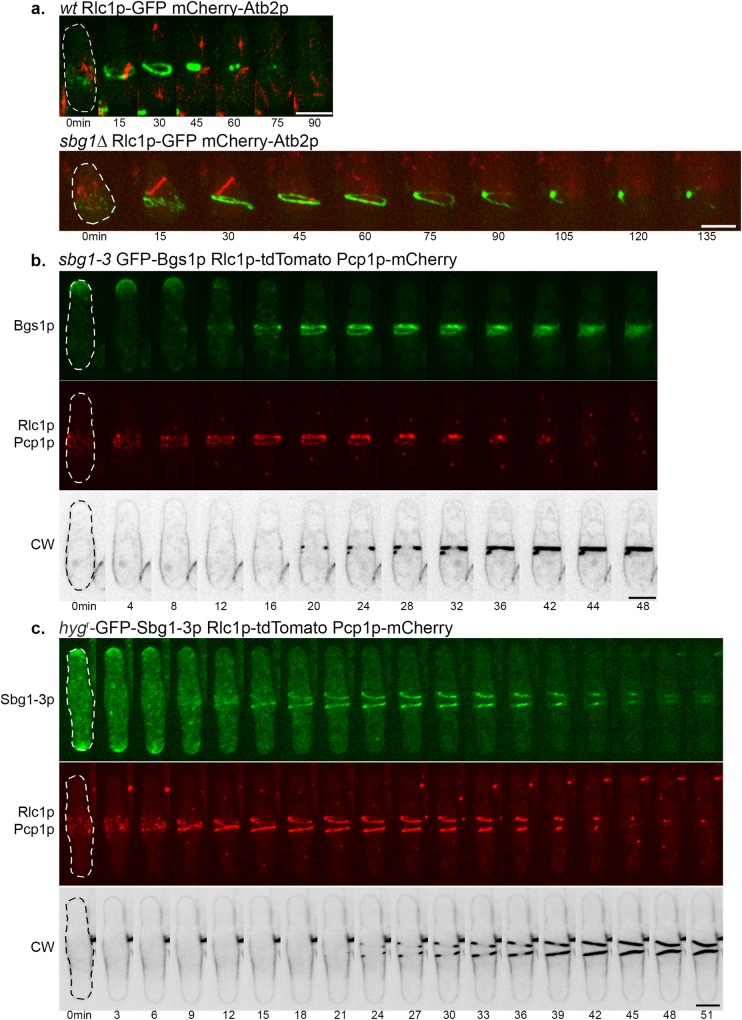
Actomyosin ring dynamics in *sbg1* mutants. (A) Maximum Z projection spinning disk confocal images of germinated cells of the indicated genotype from diploid *sbg1Δ/sbg1*^*+*^ mCherry-Atb2p Rlc1p-3GFP (MBY9156). Green, Rlc1p-GFP. Red, mCherry-Atb2p. (B) Maximum Z projection spinning disk confocal montage of the indicated strain (*sbg1-3* GFP-Bgs1p Rlc1p-tdTomato Pcp1p-mCherry—MBY9390) after 6 hr at 36°C. Green, GFP-Bgs1p. Red, Rlc1p-tdTomato Pcp1p-mCherry. Calcofluor White (CW) images were acquired as single medial plane images and are inverted for fluorescence. 0min indicates time of spindle body duplication. Defects observed in 29.1%±7.9% cells (n = 3, at least 45 cells). (C) Maximum Z projection spinning disk confocal montage of the indicated strain (*hyg*^*r*^-GFP-Sbg1-3p GFP-Bgs1p Rlc1p-tdTomato Pcp1p-mCherry—MBY9389) after 6 hr at 36°C. Green, *hyg*^*r*^-GFP-Sbg1-3p. Red, Rlc1p-tdTomato Pcp1p-mCherry. Calcofluor White (CW) images were acquired as single medial plane images and are inverted for fluorescence. 0min indicates time of spindle body duplication. Defects observed in 37.4%±6.7% cells (n = 2, at least 20 cells). Scale bar 5μm.

Recent studies have shown that the division septum provides an anchor to the actomyosin ring and affects the integrity of the ring [8,28,35,55]. Our findings established that Sbg1p was involved in primary septum formation in association with Bgs1p, and that complete loss of Sbg1p resulted in failure to synthesize a division septum. We investigated if the inability of *sbg1Δ* to septate, and to localize Bgs1p to the cell middle might have an effect on the actomyosin ring dynamics. We observed germinating wild type populations expressing Rlc1p-GFP mCherry-Atb2p as control. Wild type spores were seen to assemble an actomyosin ring, which constricted over time and resulted in successful septation (Fig 7A). Likewise, actomyosin rings were found to assemble properly in the germinating *sbg1Δ* Rlc1p-GFP mCherry-Atb2p spores. However, in 72% *sbg1Δ* Rlc1p-GFP mCherry-Atb2p germinating spores, the actomyosin ring and the spindle disassembled over time (Fig 7A). Also, the ring was never observed to constrict or guide synthesis of a division septum. This suggested that like *cps1-191* cells, *sbg1Δ* cells were capable of assembling an actomyosin ring but failed in septum synthesis. In the absence of functional Sbg1p, medial localization of Bgs1p and rigid support from an ingressing septum, the actomyosin ring dismantled eventually in *sbg1Δ* cells.

We next imaged the actomyosin ring in *sbg1-3* mutant, which assembles split and intertwined septa, to test if the actomyosin ring defects were specific to germinating *sbg1Δ* spores. The *sbg1-3* cells expressed GFP-Bgs1p and Rlc1p-tdTomato to analyze septum and ring structures respectively and also expressed Pcp1p-mCherry to follow mitotic progression. The cells were stained with CW to enable direct visualization of the septum. We observed that the ring did not constrict as a single unit in 29% of these cells (Fig 7B). We analyzed these rings as 3D projections and tilts using Imaris Bitplane (S3A Fig). We observed that the rings were intertwined and twisted and not resolved into one full compact ring. As these defective rings constricted, they guided the synthesis of similarly defective septa, as judged by CW staining. Given that Sbg1p and Bgs1p physically interact, we also wanted to determine the localization of Bgs1p in *sbg1-3*. Interestingly, Bgs1p was also observed to localize to these defective septa, suggesting its direct involvement in the synthesis of these defective septa. Similar to 3D projections of the actomyosin ring, projections showed that Bgs1p was present as two closely spaced structures corresponding to the aberrant septa that were synthesized in the cells.

In wild type cells, the fluorescent signal for Sbg1p was detected in the vicinity of the actomyosin ring and showed an overlap with Bgs1p signal. We considered if in *sbg1-3* cells, the mutant Sbg1-3p protein localized to the vicinity of the intertwined and twisted actomyosin ring. Using marker fusion tagging [47], we generated a strain that expressed Sbg1-3p mutant protein tagged in-frame with GFP at the N terminus, and also expressed Rlc1p-tdTomato and Pcp1p-mCherry. At the restrictive temperature of 36°C, we observed that the mutant Sbg1-3p protein also localized at the defective septa (Fig 7C). These images were also analyzed as 3D projections and presented an intertwined actomyosin ring and a split Sbg1-3p disc (S3B Fig). Thus, the temperature-sensitive mutant *sbg1-3* deposited aberrant septa guided by actomyosin rings that were entangled and the mutant protein itself mislocalized to the defective structures. This suggested that Sbg1p is involved in maintaining the integrity of the actomyosin ring in addition to its major role in septum synthesis.

### Sbg1p interacts with Rga7p and Imp2p

**Fig 8:**
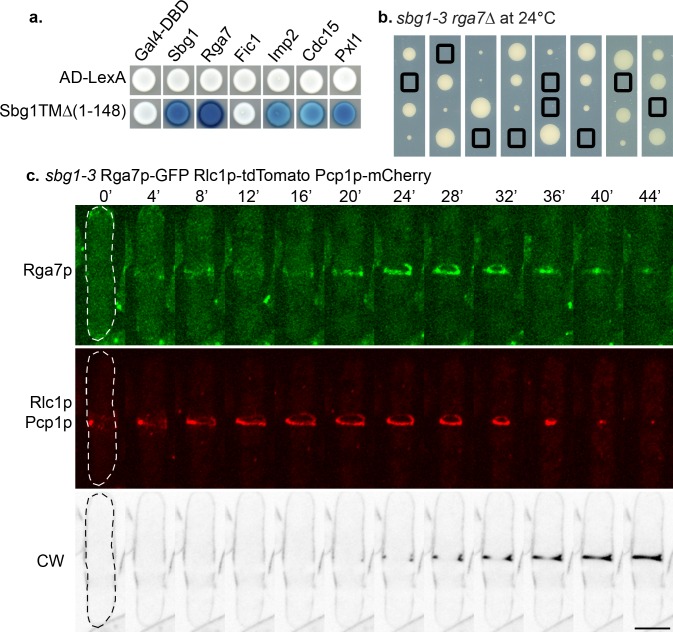
Sbg1p interacts with other ring proteins. (A) Summary of yeast two hybrid interaction of truncated Sbg1TMΔp with the indicated proteins. (B) Tetrad dissection analysis of a cross between *sbg1-3* and *rga7Δ*. Boxes indicate double mutant *sbg1-3 rga7Δ*. (C) Maximum Z projection spinning disk confocal montage of the indicated strain (*sbg1-3* Rga7p-GFP Rlc1p-tdTomato Pcp1p-mCherry—MBY9485) after 6 hr at 36°C. Green, Rga7p-GFP. Red, Rlc1p-tdTomato Pcp1p-mCherry. Calcofluor White (CW) images were acquired as single medial plane images and are inverted for fluorescence. 0min indicates time of spindle body duplication. Scale bar 5μm.

The accumulating evidence pointed towards a role for Sbg1p in preserving integrity of the actomyosin ring and linking it to the septum synthesis machinery. Absence of such an essential link (as in *sbg1Δ)* led to the unraveling of the actomyosin rings and defective septum synthesis. On the other hand, in the *sbg1-3* mutant, actomyosin ring integrity was affected, resulting in formation of double actomyosin rings and aberrant septum deposition. The phenotypic defect of intertwined double actomyosin rings in *sbg1-3* was similar to the phenotype observed in cells lacking Pxl1p, a LIM-domain containing ring protein which cooperates with Bgs1p in septum formation [35]. *pxl1Δ* cells displayed splitting of the ring into two rings at late anaphase, of which only one constricted and guided septum synthesis [34,35,42]. It has also been reported that the actomyosin ring is not well anchored in *pxl1Δ*, and slides off along the cortex towards the cell end, leading to a large proportion of cells with off-center septa [35]. Further, Pxl1p physically interacts with the two F-BAR proteins Cdc15p and Rga7p and also with Rlc1p [33,36,42]. Recent studies have implicated a role for the F-BAR proteins Cdc15p, Imp2p and Rga7p in the integrity of the ring as well [33,36,37]. Among these three F-BAR proteins, Imp2p and Rga7p appear at the cell division much later than Cdc15p. Since *sbg1-3* mutant cells displayed intertwined and twisted actomyosin rings, we assessed if Sbg1p interacted with any of these proteins using the yeast two-hybrid approach (Fig 8A). To avoid complications arising from the fact that Sbg1p is an integral membrane protein, we analyzed interactions of these proteins with a truncation of Sbg1p without the transmembrane domain (Sbg1 TMΔ: expressing residues 1–148). Interestingly, Sbg1p interacted with itself and also displayed interactions with all the proteins tested–Rga7p, Imp2p, Cdc15p and Pxl1p. We did not detect any interaction between Sbg1p and the C2-domain protein Fic1p.

In genetic crosses, we uncovered strong genetic interactions of *sbg1* with *rga7*, *pxl1* and *imp2*. The double mutants *sbg1-3 rga7Δ* (Fig 8B) and *sbg1-3 pxl1Δ* (S4C Fig) were synthetic lethal even at permissive temperature. We also observed synthetic growth defects in the double mutant *sbg1-3 imp2Δ* (S4A and S4B Fig). These results suggested the existence of a possible cooperation between Sbg1p and this network of proteins that ensure efficient ring anchorage and integrity while linking it to the Bgs1p-dependent septum synthesis machinery.

It has been shown in wild type cells that Rga7p localizes to the cell middle as a ring which is later detected as a disc as the actomyosin ring constricts [36,37]. As Sbg1p displayed genetic and physical interaction with Rga7p, we wanted to determine the localization of Rga7p in mutant *sbg1-3* cells. To this end, we imaged *sbg1-3* cells expressing Rga7p-GFP Rlc1p-tdTomato and Pcp1p-mCherry with CW staining. At the restrictive temperature, Rga7p localized to the cell middle at the defective intertwined actomyosin rings in the *sbg1-3* mutant background. Rga7p, like Sbg1p and Bgs1p, followed the defective actomyosin rings and contributed in defective septum synthesis (Fig 8C). Given the synthetic lethality of the double mutant *sbg1-3 pxl1Δ*, we also checked the localization of Pxl1p in *sbg1-3* mutant. Indeed, Pxl1p signal was also observed in the entangled rings (S4D Fig). As the actomyosin ring constricted, Pxl1p ring was also seen to constrict with it. Taken together, we conclude that Sbg1p forms an essential functional link between the actomyosin ring and the Bgs1p-dependent septum synthesis machinery in fission yeast.

## Discussion

Successful cytokinesis in many eukaryotes requires intricate coordination between the actomyosin ring and its interactions with the plasma membrane and the septum synthesis machinery [8,28,35,41]. Our work here identifies a novel physical link between these modules through the characterization of the novel integral membrane protein Sbg1p.

Sbg1p, the protein product of *SPBP22H7*.03 or *sbg1*, is an integral membrane protein. The *S*. *pombe* database suggests that the Sbg1p carries a truncated SKN1 domain within the transmembrane domain. Sbg1p shows similarity to uncharacterized β-glucan synthesis associated proteins from other Ascomycetes (e.g. *Schizosaccharomyces japonicus)*. In budding yeast, the two SKN1 proteins, Skn1p and Kre6p, are functionally redundant [56]. Loss of ScKre6p shows reduced levels of both β-1,3-glucan and β-1,6-glucan synthesis [57] while loss of Skn1p affects sphingolipid biosynthesis [58]. It will be interesting to determine if other fungal proteins perform similar functions as *S*. *pombe* Sbg1p in cytokinesis.

How does Sbg1p suppress *cps1-191*? *cps1*-*191* mutants are defective in division septum assembly and arrest the cell cycle as binucleate cells with an unconstricted actomyosin ring that slides along the cell periphery. Previous work has shown that the product of the *cps1*-*191* mutant (Cps1-191p) fails to localize properly to the vicinity of the stable actomyosin rings [35]. Overproduction of Sbg1p allows *cps1*-*191* to form colonies at 34°C, but not at 36°C, suggesting that Sbg1p is unlikely to be a bypass suppressor of *cps1*-*191*, but one that increases the activity of the partially compromised Cps1-191p. It appears that excess Sbg1p, itself a protein that localizes to the actomyosin ring like Bgs1p and physically interacts with Bgs1p, facilitates medial localization of Cps1-191p leading to actomyosin ring retention and constriction at the division site and more efficient primary septum assembly. Interestingly, overproduced Sbg1p localized to the division site in *cps1-191* cells at 34°C whereas endogenous levels of Sbg1p failed to do so in *cps1-191* mutant background. Consistent with the recent proposals, it is likely that Bgs1p is a tension (stretch) activated protein [13,59], and its presence in the actomyosin ring in the rescued cells would allow tension generated by the actomyosin ring to activate Cps1-191p leading to primary septum assembly.

What is the physiological function of Sbg1p? Deletion of *sbg1* leads to lethality concomitant with a defect in polarized growth and division septum assembly. *sbg1Δ* cells are therefore large and rounded in morphology and accumulate more than 2 nuclei. We have shown that Sbg1p and Bgs1p show physical interactions, although it is presently unknown if these interactions are direct or indirect or if Bsg1p and Sbg1p simply reside in the same membrane compartment. The buffer conditions we have used have been used to report a physical interaction between Bgs1p and Ags1p [28]. In light of this, the localization dependencies and the similar physiological roles of Bgs1p and Sbg1p in septation and β-1,3-glucan synthesis, we propose that Sbg1p physically interacts with Bgs1p. It is likely that Sbg1p plays a key role in the stable localization and function of Bgs1p at the division site. This in turn potentially leads to actomyosin ring tension dependent activation of Bgs1p synthase activity and primary septum assembly. Sbg1p is also detected at the nuclear envelope (NE) and the endoplasmic reticulum (ER) like strands. It is unclear if it plays a role in the NE/ER or if its presence in the NE/ER simply reflects its path in biogenesis.

**Fig 9:**
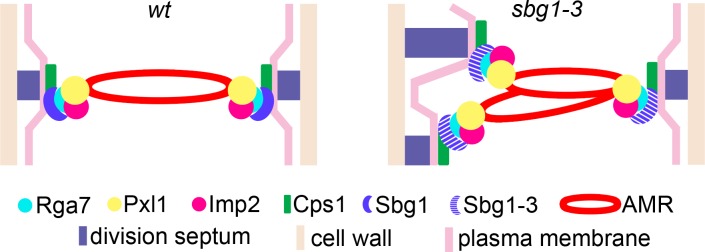
Sbg1p functions as a critical link between contractile ring apparatus and the septum synthesis machinery. Illustration depicting interaction of Sbg1p with components of the actomyosin ring and Bgs1p-dependent septum synthesis machinery. Disruption in function of Sbg1p in the mutant *sbg1-3* results in intertwined and twisted actomyosin rings which guide aberrant or altered septum synthesis.

*sbg1Δ* cells assemble actomyosin rings that dismantle, rather than constrict, leading to a full failure to synthesize primary septa. By contrast, the temperature-sensitive mutant, *sbg1-3*, shows defects in actomyosin ring integrity and stability leading to split / unraveled actomyosin rings that make a correspondingly split / unraveled primary septa (Fig 9). The strong defect of *sbg1Δ* in actomyosin ring stability suggests that in addition to a role in primary septum assembly, Sbg1p may have a direct role in actomyosin ring stability / function. The fact that actomyosin rings are not stably maintained at the division site in *sbg1-3*, suggests that Sbg1p may interact with the plasma membrane and Bgs1p on the one hand and with actomyosin ring proteins on the other (Fig 9). Thus Sbg1p may be a link between the actomyosin ring and the plasma membrane and Bgs1p-dependent septum synthesis machinery.

How does Sbg1p regulate actomyosin ring function? In wild type cells, the contractile ring is linked to the membrane by the F-BAR proteins Cdc15p, Imp2p and Rga7p [32,35,36]. Rga7p directly interacts with Pxl1p and Imp2p, and further Pxl1p interacts with Cdc15p and Rlc1p at the ring itself [36,42]. We have found genetic and / or physical interactions (through yeast two hybrid strategy) of Sbg1p with Imp2p, Pxl1p, Cdc15p and Rga7p. Thus, Sbg1p is a new component amongst a group of actomyosin ring proteins that regulate proper septum assembly as well as regulate actomyosin ring integrity. We propose that Sbg1p could potentially function as a link between the contractile elements of the actomyosin ring (F-actin, tropomyosin, myosin II, formin, α-actinin) with the machinery that assembles the primary septum (Bgs1p and possibly Bgs4p and Ags1p), which are also involved in assembly of the primary septum. Key ring proteins known to be involved in maintaining the integrity of actomyosin ring include Cdc15p, Imp2p, Rga7p, Pxl1p, and Sbg1p. Of these, Sbg1p, the only integral membrane protein in this group, requires its membrane spanning sequence for function, consistent with a potential role as a critical link between the plasma membrane and the actomyosin ring.

Interestingly, a recent study in budding yeast unveiled the presence of an Ingression Progression Complex (IPC) which regulates coordinated actomyosin ring constriction, plasma membrane ingression and simultaneous septum synthesis [60]. This complex contains at least five ring proteins, Myo1p (myosin II), Iqg1p (IQGAP protein), Hof1p (F-BAR protein), Inn1p (C2 domain protein) and Cyk3p, all of which interact with Chs2p, the enzyme responsible for primary septum synthesis in budding yeast. The proteins in the IPC function in the efficient localization and activation of Chs2p at the division site. Perturbation of the function of any of the IPC proteins affects the function of Chs2p and primary septum synthesis. Though the biochemical composition of primary septum (composed of chitin) differs in budding yeast and fission yeast, orthologues of all the proteins in IPC are present in fission yeast. Whether such a complex exists in fission yeast and the identities of its core components are yet to be determined.

Through this study we have found that Sbg1p and Bgs1p are co-dependent for their localization at the division site. It would be interesting to know what triggers the localization of Sbg1p / Bgs1p to the division site. Further, we have found that Sbg1p interacts with other ring proteins that together function in actomyosin ring stabilization. It is not known if all these proteins function in a single complex *in vivo* and which proteins physically interact directly with Sbg1p. Through yeast two-hybrid analysis we also found that Sbg1p interacts with itself, although the function of this homo-dimerization remains to be determined. Future work should investigate these questions.

## Materials and Methods

### Yeast strains and methodology

A list of *S*. *pombe* strains, plasmids and primers used in this study are provided in S3 Table. Standard genetic and molecular biology protocols for *S*. *pombe* were used as described previously [61].

For the generation of *hyg*^*r*^-GFP-Sbg1p, we used a method similar to that described in Lai et al [47]. Briefly, three DNA fragments corresponding to 5’UTR of *sbg1* ORF, *hyg*^r^-eGFP (pCDL1522) and first 540bp of *sbg1* ORF were obtained with primers MOH6061 to MOH6066.The fragments had overhangs such that the three fragments could be fused together by fusion PCR to generate 5’UTR_*sbg1-hyg*^r^-eGFP-ORF_*sbg1*. This PCR fragment was then transformed in wild type cells to obtain the *hyg*^*r*^-GFP-Sbg1p strain by selection for hygromycin resistance and by sequencing.

### Plasmids and recombinant DNA methods

For generation of pSbg1-TMΔ (pBac14), two PCR fragments were generated—fragment 1 (primers K150 and K151) to amplify a 396bp fragment from +55 to +450 of *sbg1*^*+*^ ORF, fragment 2 (primers K152 and K153) to amplify a 487bp fragment starting from +517 of ORF and extending into the 3’UTR of *sbg1*. The fragments had overhangs allowing fusion PCR to generate a DNA fragment corresponding to *sbg1* ORF without the TM domain. This fragment was then used for inverse PCR with pSbg1 (pBac5), to delete the TM domain from the plasmid.

For the experiment with GFP-Cps1-191p, we modified plasmid pSbg1 (pBac5) and pCDL1000 to edit out the leucine expressing fragment and replaced it with a histidine expressing fragment. These plasmids are referred to as pSbg1_his3 (pBac7) and pEmpty_his3 (pBac6), respectively.

For generation of p*mCh-sbg1*^*+*^ (pBac9), a PCR product (K154 and K155) was used to generate a 708bp fragment using pCDL1687 as a template. The PCR fragment had overhangs at the mCherry sequence to allow for fusion at the N-terminus of *sbg1*^*+*^ on plasmid pBac7. The fusion was done by inverse PCR.

### Microscopy

For comparing the fluorescence intensity of Calcofluor White at the septum in two strains for Fig 2C, CW was used at a final concentration of 10 μg/ml (Stock concentration 10mg/mL) and FITC-ConA was used at a final concentration of 20 μg/ml (Stock concentration 1mg/mL) as described [28]. These images were acquired using a fluorescence microscope (model DM RXA; Leica), a PL APO 63×/1.32 oil PH3 objective, a digital camera (model DFC350FX; Leica) and CW4000 cytoFISH software (Leica). Quantification of fluorescence intensity was done as described [35].

Bright-field images were obtained with a microscope (IX71; Olympus; Plan Apochromat 100×/1.45 NA oil objective lens) equipped with a charge-coupled device camera (CoolSNAP HQ; Photometrics) and MetaMorph (v6.2r6) software (Molecular Devices). Spinning-disk images were acquired with a *micro*LAMBDA spinning disk using a microscope (Eclipse Ti; Nikon; Plan Apochromat VC 100×/1.40 NA oil objective lens) was equipped with a spinning-disk system (CSUX1FW; Yokogawa Corporation of America), camera (CoolSNAP HQ^2^), and MetaMorph (v7.7.7.0) software. A 491-nm diode-pumped solid-state (DPSS) laser (Calypso), 515-nm DPSS laser (Fandango; Cobolt), and 561-nm DPSS laser (Jive) were used for excitation. Spinning disk confocal images were acquired at 0.5μm step size, for a range of 6μm for all images except those of *sbg1Δ* which was at 10μm. All images are presented as 2D maximum intensity projection. Image processing was done using ImageJ (v1.47).

### Transmission electron microscopy

Cells were prepared for TEM as described in [35,62,63]. Briefly, cells were fixed with 2% glutaraldehyde EM (GA; Electron Microscopy Science) in 50 mM phosphate buffer pH 7.2, 150 mM NaCl (PBS) for 2 h at 4°C, post-fixed with 1.2% potassium permanganate overnight at 4°C. Cells were then embedded in 2% low-melting-point agarose, dehydrated through an ethanol series, and passed through QY-2 (methyl glycidyl ether; Nisshin EM, Tokyo, Japan). Next, cells were embedded in Quetol 812 mixture (Nisshin EM Tokyo, Japan). Ultrathin sections were stained in 4% uranyl acetate and 0.4% lead citrate, and viewed with a TEM JEM-1400 (JEOL, Tokyo, Japan) at 100 kV.

### Multi-copy suppressor screen of *cps1-191*

The *cps1-191* mutant was transformed with *S*. *pombe* genomic libraries, pTN-L1 [64]. Transformants were selected from selective minimal plates lacking leucine at 24°C. These were replicated to YES plates with Phloxin B at 34°C. Plasmids were isolated from viable colonies and sequenced (Primers MOH1207 and MOH1208) to identify the gene fragment responsible for rescue of *cps1-191* lethality.

### Immunoprecipitation and immunoblot analysis

Immunoprecipitation experiments were performed similar to that described in [28]. Briefly cells from indicated strains were harvested and washed with stop solution (154 mM NaCl, 10 mM EDTA, 10 mM NaN_3_, and 10 mM NaF), then with wash buffer (50 mM Tris-HCl, pH 7.5, 5 mM EDTA). Cells were then lysed by glass beads in lysis buffer (50 mM Tris-HCl pH 7.5, 5 mM EDTA, 200 mM NaCl containing 100 μM phenylmethylsulphonylfluoride, 1mM benzamidine and protease inhibitors—Complete EDTA-free, Roche Diagnostics). Cell debris was removed by centrifugation (4,500 *g*, 1 min, 4°C). The supernatant was used to obtain cell membranes by centrifugation (16,000 *g*, 1 hr, 4°C). The cell membrane pellet thus obtained was resuspended in immunoprecipitation buffer (IPB; 50 mM Tris-HCl, pH 7.5, 5 mM EDTA, 200 mM NaCl, 0.5% Tween 20, 100 μM phenylmethylsulphonylfluoride, 1mM benzamidine and protease inhibitors—Complete EDTA-free, Roche Diagnostics), and agitated (1,300 rpm, 30 min, 1°C; Thermomixer Comfort, Eppendorf). This agitated suspension was centrifuged (21,000 *g*, 30 min, 4°C); supernatant was collected, diluted with IPB, and the solubilized membrane proteins were incubated with antibody against GFP (Abcam) for 1 hr at 4°C. Sepharose protein A beads were added to this mix for 3 hr, later washed with IPB and boiled in sample buffer. Whole cell lysate and IPs were then resolved on 4%-20% gels (Biorad) transferred to Immobilon-P membrane (Millipore), blocked and immunoblotted using antibodies against GFP (1:2500, Abcam) or HA (1:5000, Roche Diagnostics). Peroxidase conjugated -rabbit or -mouse secondary antibodies (Jackson Laboratories) were used at 1:20000 dilutions. Signals were detected using enhanced chemiluminescence.

### Yeast cell lysis

Yeast cell lysis was performed as described previously [65,66]. Cells at 0.8–1 OD were collected and resuspended in 800μL of cold water. This cell suspension was mixed with 150μl of freshly prepared buffer (1.85M NaOH, 7.5% β-mercaptoethanol) and left on ice for 10 min. Next, 150μl of 55% trichloroacetic acid (TCA) (w/v, stored in the dark) was added to the mixture and left for another 10–15 min on ice. The mix was then centrifuged at 20 min at 15,000 rpm at 4°C, supernatant was discarded. The pelleted cells were briefly centrifuged again, and all traces of TCA were removed. The pellet was then resuspended in 50μl HU-buffer [65]. The proteins were denatured at 95°C for 5min or 65°C for 15min. Finally the protein samples were resolved by SDS-PAGE and immnuoblotted as described in the IP protocol.

### Marker reconstitution mutagenesis

Marker reconstitution mutagenesis was used to screen for *sbg1* mutants as described [52]. Two PCR fragments, with K47 and K48 and with K49 and K50 primers, were obtained. These two fragments were fused by PCR and then digested with *Spe*I and *Sal*I and subcloned into pCDL1542 digested at *Sal*I-*Nhe*I site. The plasmid thus obtained was digested with *Pvu*II and then transformed into MBY6218. Positive transformants were selected on minimal media plates lacking uracil and were confirmed by sequencing to obtain strain MBY9198. A PCR fragment was obtained with K140 and K141 and used for inverse PCR into pCDL1456 to generate plasmid pCDL1673. Using primers K185 and K186 mutagenesis PCR was performed to obtain fragment *sbg1-his5c*^+^ which was transformed into MBY9198. Colonies were selected on minimal medium plates lacking histidine at 24°C and then replicated to YES plates containing Phloxin B at 36°C. Mutants were screened as colonies showing dark red on YES with Phloxin B at 36°C. Mutants were sequenced for *sbg1* gene and also backcrossed to ensure mutations were tightly linked to *his5*^*+*^ and *ura4*^*+*^.

### Yeast two-hybrid

Yeast two-hybrid analyses were performed as described [67,68]. Indicated genes or fragments were cloned into pMM5 and pMM6 plasmids. Plasmids were transformed into SGY37 (MATa) and YPH500 (MATalpha) yeast strains.

## Supporting Information

S1 Fig Multi-copy expression of *sbg1*^*+*^ improves quality of septum synthesized in *cps1-191* cells. (A) Quantification of the displacement of the septum from the cell middle in the indicated strains: wt+pEmpty (MBY8558) and *cps1-191+*pSbg1 (MBY8946) after 16 hr at 34°C. (B) Quantification of the type of septa observed with 3D analysis of confocal spinning disk images of the indicated strains: wt+pEmpty (MBY8558), *cps1-191+*pEmpty (MBY8944) and *cps1-191+*pSbg1 (MBY8946) after 16 hr at 34°C.
(TIF)

S2 Fig Sbg1p is an essential integral membrane protein. (A) Cells from strain *hyg*^*r*^-GFP-Sbg1p HA-Bgs1p (MBY9241) were lysed and proteins extracted in lysis buffer containing the compounds indicated. This was subjected to ultra-centrifugation and the supernatant (S) and pellet (P) fractions from each treatment were resolved using SDS-PAGE gels and immunoblotted using monoclonal anti-HA antibodies and monoclonal anti-GFP antibodies. (B) Bgs1p physically interacts with Sbg1p. Solubilized membrane proteins from the indicated strains: wt (MBY192), Ags1p- GFP HA-Bgs1p (MBY8674), *hyg*^*r*^-GFP-Sbg1p (MBY8967) and HA-Bgs1p *hyg*^*r*^-GFP-Sbg1p (MBY9241) were immunoprecipitated (IP) with anti-GFP antibodies. Solubilized membrane proteins (input, top) and IP (bottom) were transferred to the same membrane and blotted with monoclonal anti-HA antibodies. (C) Tetrad dissection analysis of diploid *sbg1Δ/sbg1*^*+*^ (MBY9086) showing 2:2 segregation of growth on YES plates. Images show multinucleated germinated *sbg1Δ* spores.
(TIF)

S3 Fig Analysis of actomyosin ring and septum synthesis machinery in *sbg1-3* cells. (A) 3D projection and 3D projection tilt images obtained with Imaris for Fig 7B. Green: GFP-Bgs1p, Red: Rlc1p-tdTomato, Pcp1p-mCherry. 0min indicates time of spindle body duplication. (B) 3D projection and 3D projection tilt images obtained with Imaris for Fig 7C. Green: *hyg*^*r*^-GFP-Sbg1-3p, Red: Rlc1p-tdTomato, Pcp1p-mCherry. 0min indicates time of spindle body duplication. Scale bar 5μm.
(TIF)

S4 Fig Genetic interaction of *sbg1* with *imp2* and *pxl1*. (A) Image of a petridish showing very slow growth of the double mutant *sbg1-3 imp2Δ* (MBY9400) as compared to both single mutants *imp2Δ* (MBY737), *sbg1-3* (MBY9359) and wild type cells (MBY192) at 24°C. (B) Calcofluor white (CW) images of the medial plane of fixed cells from the indicated strains: wt (MBY192), *imp2Δ* (MBY737), *sbg1-3* (MBY9359) and *imp2Δ sbg1*-3 (MBY9400) at 24°C. (C) Tetrad dissection analysis of a cross between *sbg1-3* and *pxl1Δ*. Boxes indicate double mutant *sbg1-3 pxl1Δ*. (D) Maximum z projection spinning disk confocal montage of the indicated strain (*sbg1-3* GFP-Pxl1p Rlc1p-tdTom Pcp1p-mCherry—MBY9448) after 6 hr at 36°C. Green, GFP-Pxl1p. Red, Rlc1p-tdTomato Pcp1p-mCherry. Calcofluor White (CW) images were acquired as single medial plane images and are inverted for fluorescence. 0min indicates time of spindle body duplication. Defect observed in 15%±9.6% cells (n = 2, at least 50 cells). Scale bar 5μm.
(TIF)

S1 Table Cell wall fractionation values of the indicated strains at 24°C and after 16 hr at 34°C. Numbers in parentheses indicate percentage of each component in total cell wall. Student’s t-test was performed for the percentage of each polysaccharide in the cell wall for the combinations indicated in the lower table.
(TIF)

S2 Table Cell wall fractionation values of the indicated strains at 24°C, after 4.5 hr (*cps1-191*) or 24 hr (*sbg1-3*) at 36°C. Numbers in parentheses indicate percentage of each component in total cell wall.
(TIF)

S3 Table *S*. *pombe* strains used in this study. (PDF)

S4 Table Yeast strains used for yeast two hybrid analysis (PDF)

S5 Table List of plasmids used in this study. (PDF)

S6 Table List of primers used in this study. (PDF)

S1 Text Supplementary data. (DOCX)
