# Supplementary figures and images for: A New Membrane Protein Sbg1 Links the Contractile Ring Apparatus and Septum Synthesis Machinery in Fission Yeast
Source: PLoS Genet. 2016 Oct 17;12(10):e1006383. doi: 10.1371/journal.pgen.1006383 (PMC5066963; doi:10.1371/journal.pgen.1006383)

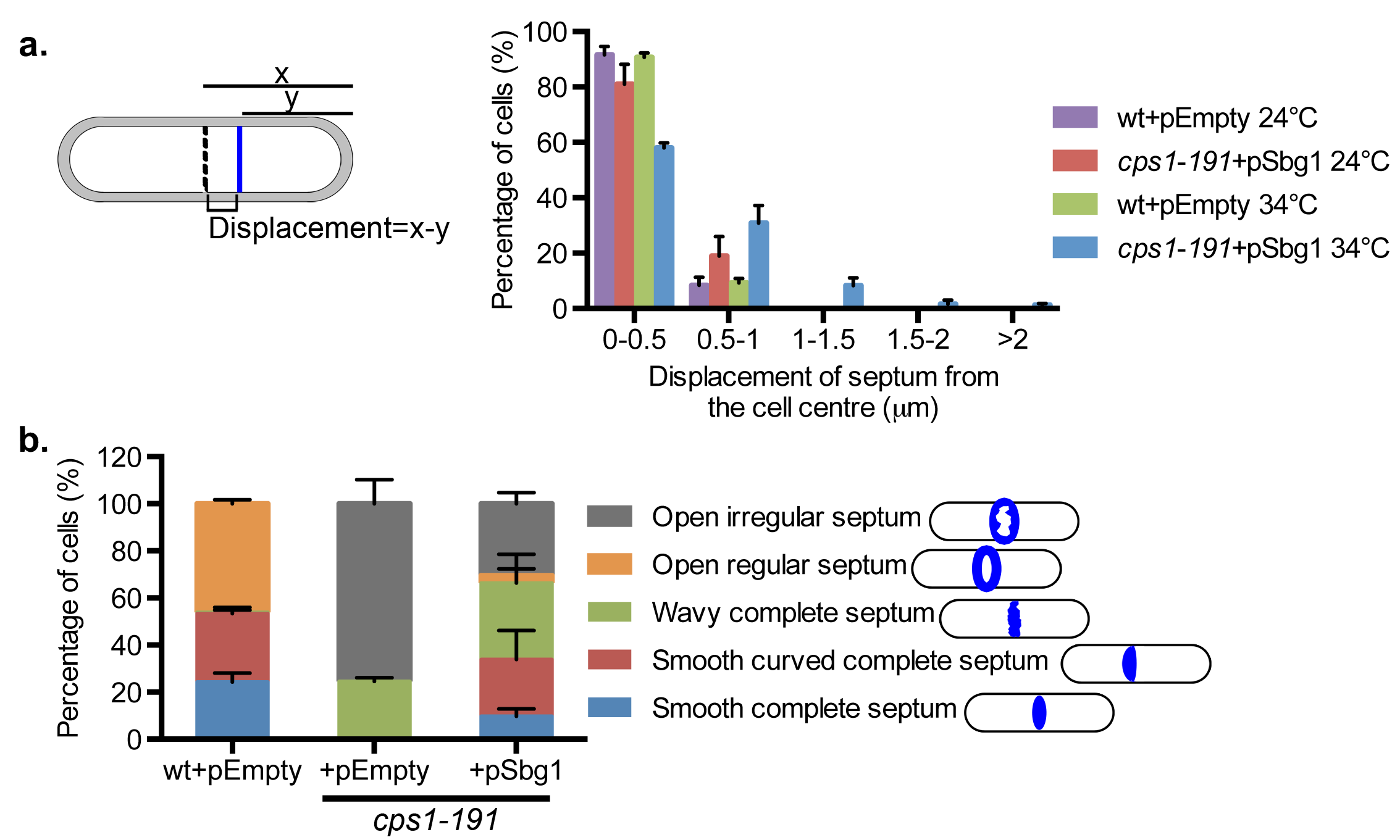

Supplement: S1 Fig — (A) Quantification of the displacement of the septum from the cell middle in the indicated strains: wt+pEmpty (MBY8558) and cps1-191+pSbg1 (MBY8946) after 16 hr at 34°C. (B) Quantification of the type of septa observed with 3D analysis of confocal spinning disk images of the indicated strains: wt+pEmpty (MBY8558), cps1-191+pEmpty (MBY8944) and cps1-191+pSbg1 (MBY8946) after 16 hr at 34°C. (TIF) [file pgen.1006383.s001.tif]

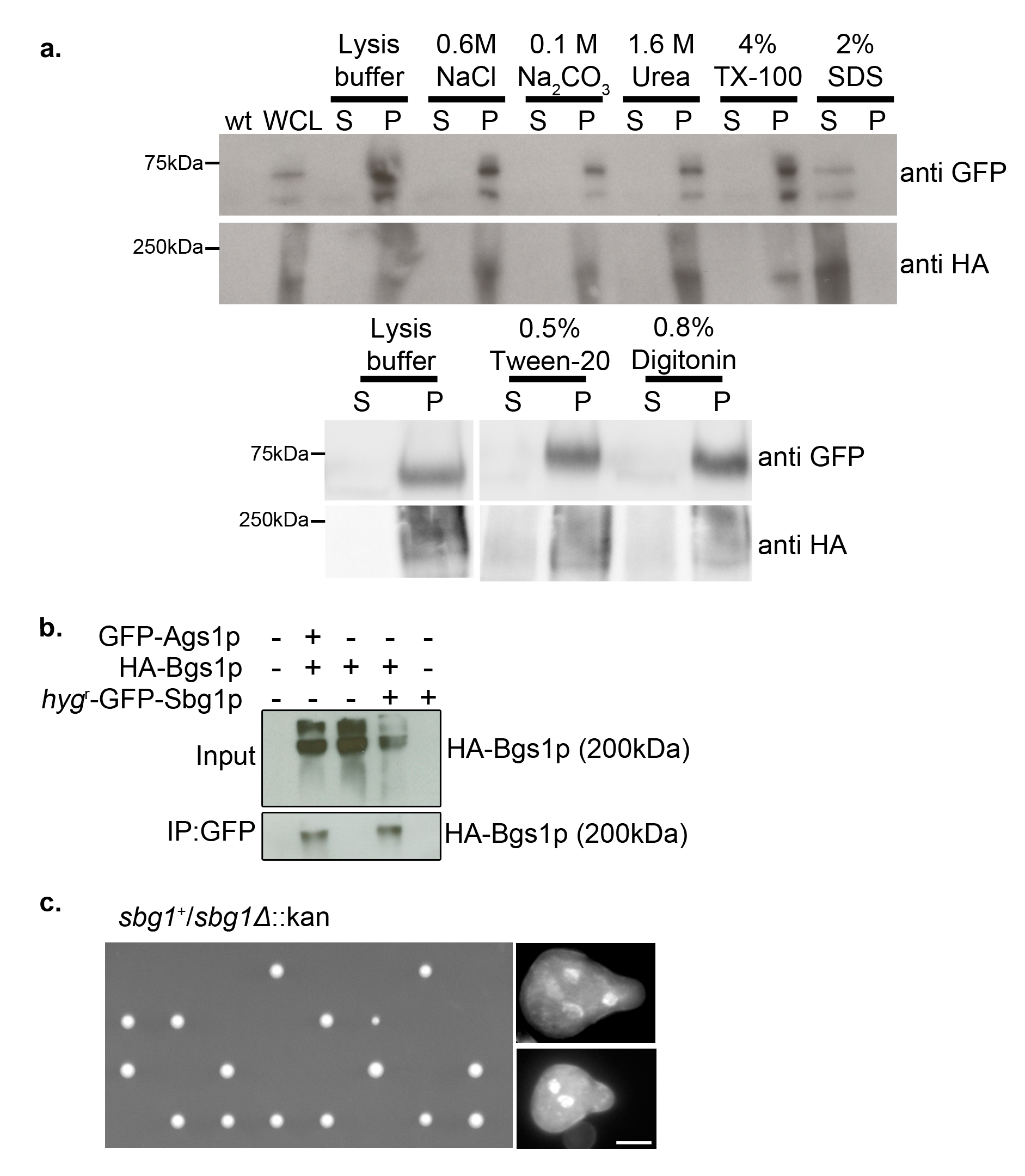

Supplement: S2 Fig — (A) Cells from strain hygr-GFP-Sbg1p HA-Bgs1p (MBY9241) were lysed and proteins extracted in lysis buffer containing the compounds indicated. This was subjected to ultra-centrifugation and the supernatant (S) and pellet (P) fractions from each treatment were resolved using SDS-PAGE gels and immunoblotted using monoclonal anti-HA antibodies and monoclonal anti-GFP antibodies. (B) Bgs1p physically interacts with Sbg1p. Solubilized membrane proteins from the indicated strains: wt (MBY192), Ags1p- GFP HA-Bgs1p (MBY8674), hygr-GFP-Sbg1p (MBY8967) and HA-Bgs1p hygr-GFP-Sbg1p (MBY9241) were immunoprecipitated (IP) with anti-GFP antibodies. Solubilized membrane proteins (input, top) and IP (bottom) were transferred to the same membrane and blotted with monoclonal anti-HA antibodies. (C) Tetrad dissection analysis of diploid sbg1Δ/sbg1+ (MBY9086) showing 2:2 segregation of growth on YES plates. Images show multinucleated germinated sbg1Δ spores. (TIF) [file pgen.1006383.s002.tif]

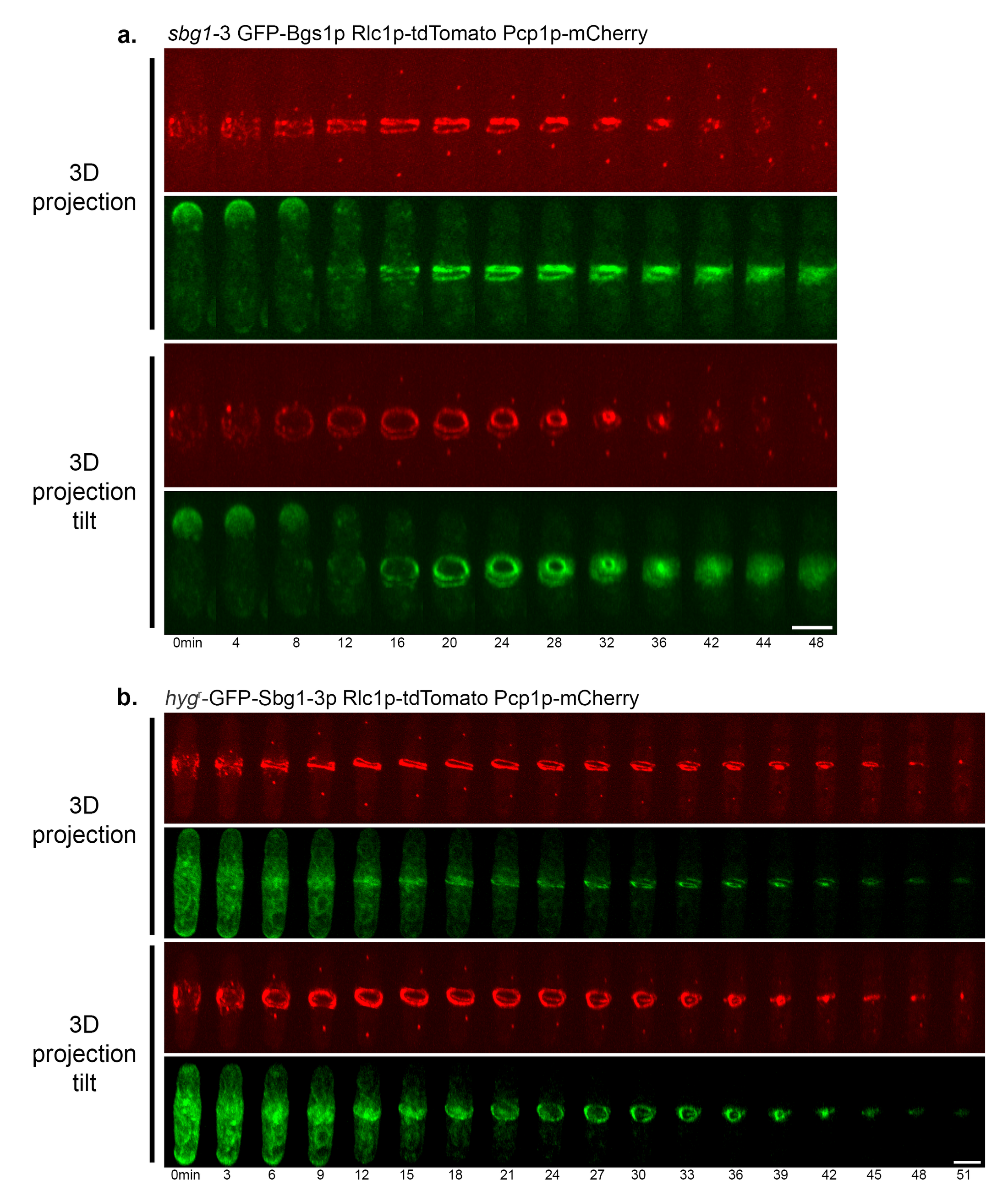

Supplement: S3 Fig — (A) 3D projection and 3D projection tilt images obtained with Imaris for Fig 7B. Green: GFP-Bgs1p, Red: Rlc1p-tdTomato, Pcp1p-mCherry. 0min indicates time of spindle body duplication. (B) 3D projection and 3D projection tilt images obtained with Imaris for Fig 7C. Green: hygr-GFP-Sbg1-3p, Red: Rlc1p-tdTomato, Pcp1p-mCherry. 0min indicates time of spindle body duplication. Scale bar 5μm. (TIF) [file pgen.1006383.s003.tif]

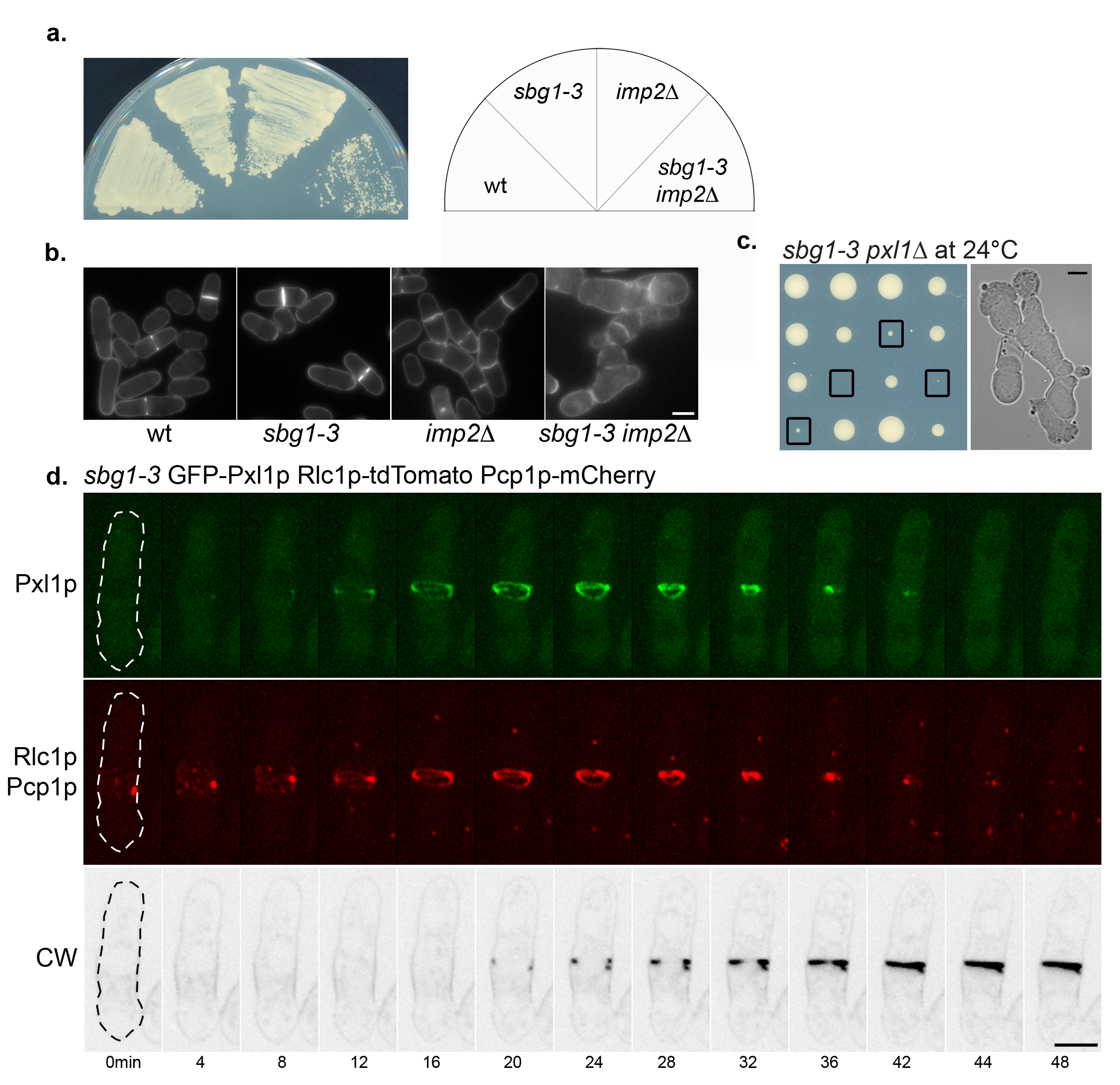

Supplement: S4 Fig — (A) Image of a petridish showing very slow growth of the double mutant sbg1-3 imp2Δ (MBY9400) as compared to both single mutants imp2Δ (MBY737), sbg1-3 (MBY9359) and wild type cells (MBY192) at 24°C. (B) Calcofluor white (CW) images of the medial plane of fixed cells from the indicated strains: wt (MBY192), imp2Δ (MBY737), sbg1-3 (MBY9359) and imp2Δ sbg1-3 (MBY9400) at 24°C. (C) Tetrad dissection analysis of a cross between sbg1-3 and pxl1Δ. Boxes indicate double mutant sbg1-3 pxl1Δ. (D) Maximum z projection spinning disk confocal montage of the indicated strain (sbg1-3 GFP-Pxl1p Rlc1p-tdTom Pcp1p-mCherry—MBY9448) after 6 hr at 36°C. Green, GFP-Pxl1p. Red, Rlc1p-tdTomato Pcp1p-mCherry. Calcofluor White (CW) images were acquired as single medial plane images and are inverted for fluorescence. 0min indicates time of spindle body duplication. Defect observed in 15%±9.6% cells (n = 2, at least 50 cells). Scale bar 5μm. (TIF) [file pgen.1006383.s004.tif]

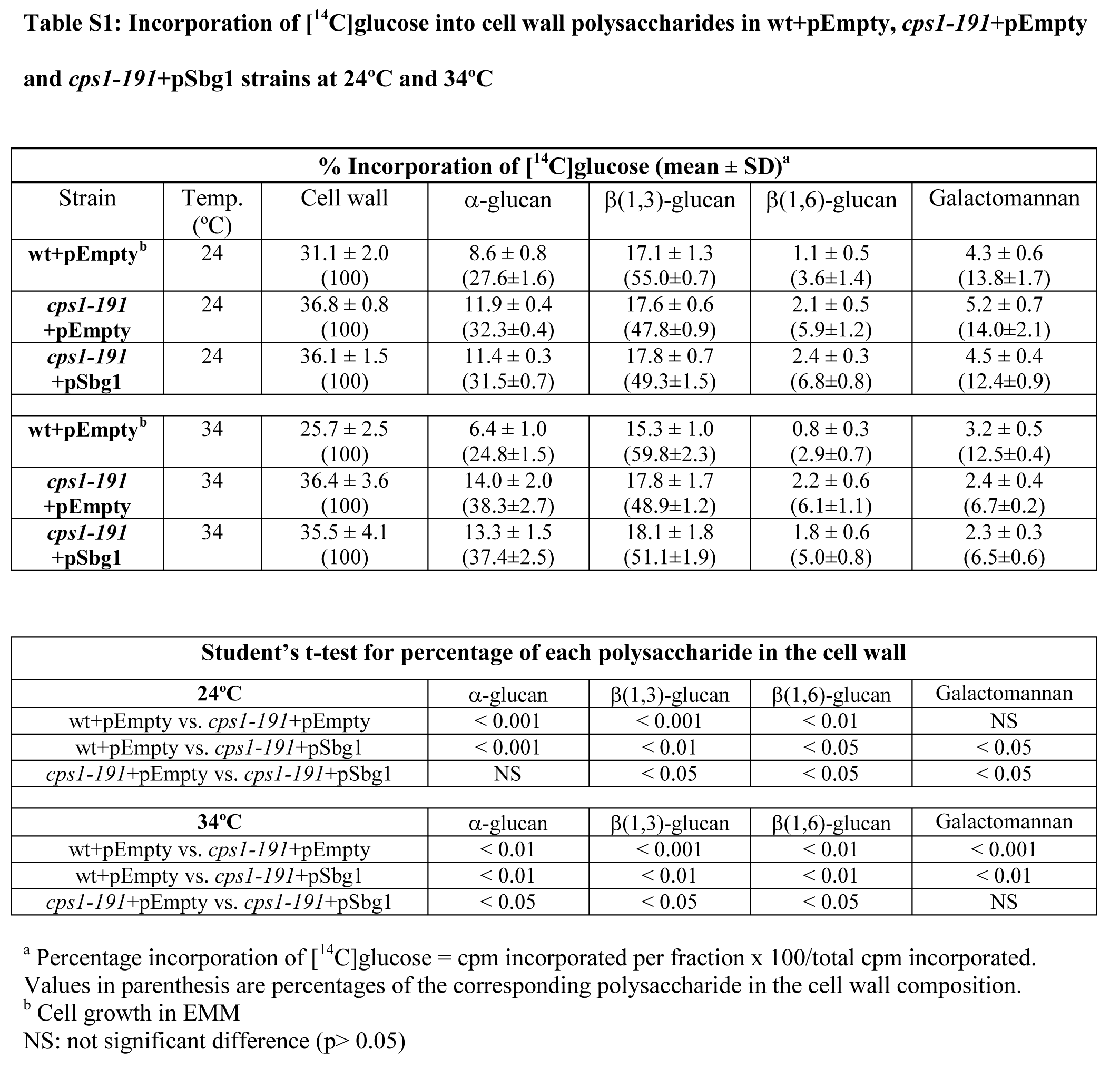

Supplement: S1 Table — Numbers in parentheses indicate percentage of each component in total cell wall. Student’s t-test was performed for the percentage of each polysaccharide in the cell wall for the combinations indicated in the lower table. (TIF) [file pgen.1006383.s005.tif]

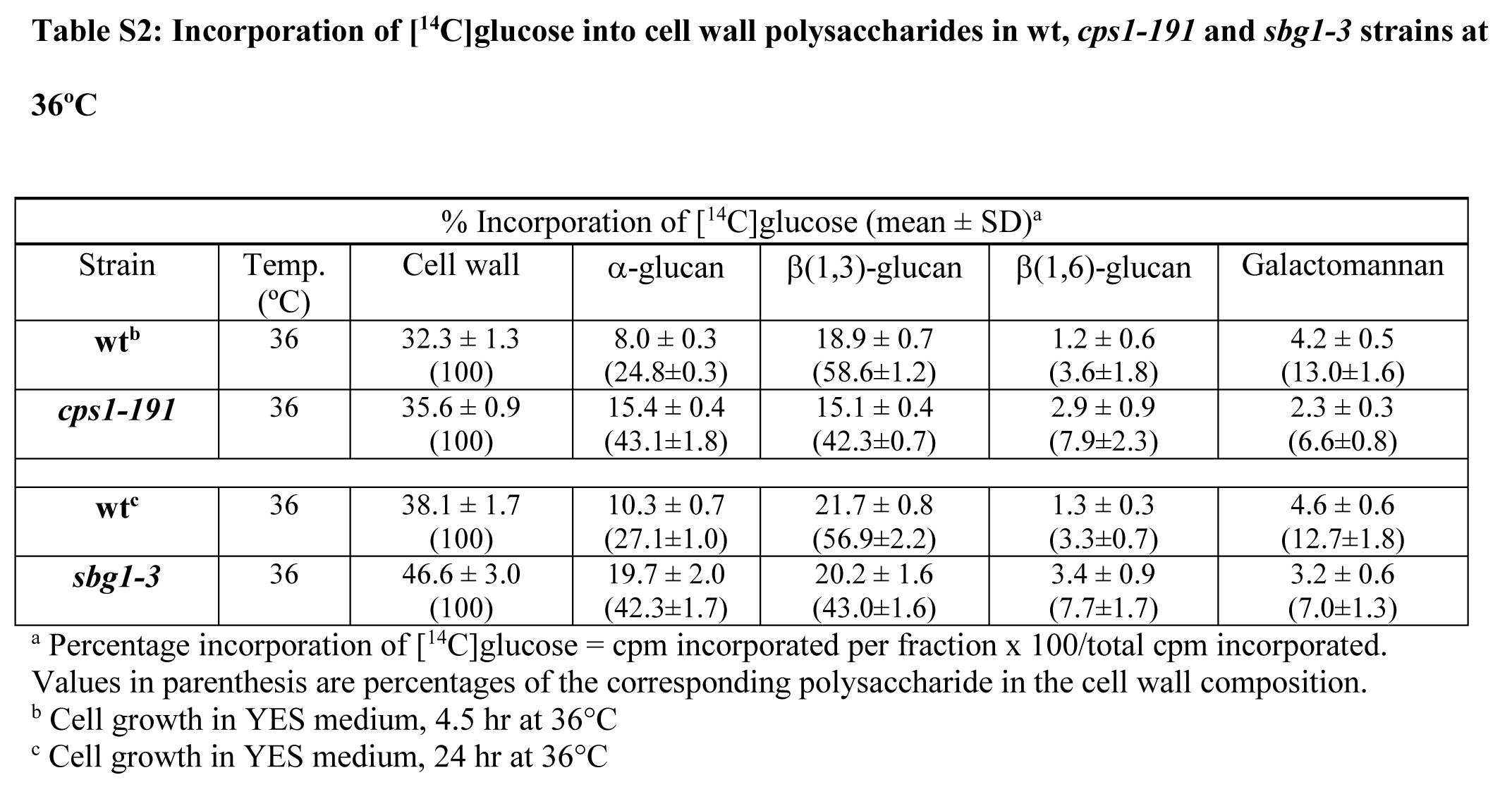

Supplement: S2 Table — Numbers in parentheses indicate percentage of each component in total cell wall. (TIF) [file pgen.1006383.s006.tif]
