# Supplementary material for: A New Membrane Protein Sbg1 Links the Contractile Ring Apparatus and Septum Synthesis Machinery in Fission Yeast
Source: PLoS Genet. 2016 Oct 17;12(10):e1006383. doi: 10.1371/journal.pgen.1006383 (PMC5066963; doi:10.1371/journal.pgen.1006383)
Supplement: S3 Table — (PDF) [file pgen.1006383.s007.pdf]

Table S3: *S. pombe* strains

MBY 102	*ade6-M210 ura4-Δ18 leu1-32* h^{+}	Lab collection
MBY 103	*ade6-M216 ura4-Δ18 leu1-32* h^{-}	Lab collection
MBY 192	*ura4-Δ18 leu1-32* h^{-}	Lab collection
MBY 737	*imp2Δ::ura4* $^{+}$ *leu1-32 ade6-M216 h*$^{+}$	Lab collection
MBY 977	*clp1Δ::ura4*$^{+}$ *ura4-Δ18 leu1-32* h^{+}	Lab collection
MBY 1148	*cps1-191 ade6-M21x ura4-Δ18 leu1-32* h^{+}	Lab collection
MBY 5730	*cps1-191 rlc1*$^{+}$*-gfp::ura*$^{+}$ *pcp1*$^{+}$*-gfp:KanMX6*	Lab collection
MBY 5732	*rlc1*$^{+}$*-gfp::ura*$^{+}$ *pcp1*$^{+}$*-gfp:KanMX6 h*$^{-}$	Lab collection
MBY 6128	*ade5Δ ade7Δ::ade5*$^{+}$ *his5Δ leu1-32 ura4-Δ18* h^{-}	Lab collection
MBY 8558	*ura4-Δ18 leu1-32 with pEmpty* h^{-}	Lab collection
MBY 8674	*ags1Δ 3'UTR*$_{ags1+}$*::ags1*$^{+}$*-GFP:leu1*$^{+}$*:ura4*$^{+}$ *bgs1Δ::ura4*$^{+}$ P_{bgs1+}*::3XHA-bgs1*$^{+}$*:leu1*$^{+}$ *leu1-32 ura4-Δ18 ade6-M21X* h^{+}	Lab collection
MBY 8702	P_{bgs1+}*::3XHA-bgs1*$^{+}$*:leu1*$^{+}$ *bgs1Δ::ura4*$^{+}$ *leu1-32 ura4-Δ18* h^{+}	Lab collection
MBY 8865	*leu1-32 ura4-Δ18 his3-Δ1 bgs1Δ::ura4*$^{+}$ P*bgs1*$^{+}$*::GFP-bgs1*$^{+}$*:leu1*$^{+}$ h^{-}	Lab collection
MBY 8944	*cps1-191 ade6-M21x ura4-Δ18 leu1-32 with pEmpty* h^{+}	This study
MBY 8946	*cps1-191 ade6-M21x ura4-Δ18 leu1-32 with pSbg1* h^{+}	This study
MBY 8947	*cps1-191 ade6-M21x ura4-Δ18 leu1-32 with pCps1* h^{+}	This study
MBY 8967	*Hyg*^{r}*:eGFP-sbg1*$^{+}$ *ura4-Δ18 leu1-32* h^{-}	This study
MBY 8977	*Hyg*^{r}*:eGFP-sbg1*$^{+}$ *mCherry-atb2*$^{+}$*:hph leu1-32 ura4-Δ18* h^{+}	This study
MBY 9006	*Hyg*^{r}*:eGFP-sbg1*$^{+}$ P_{bgs1+}*::tdTom-12A-bgs1*$^{+}$*:leu1*$^{+}$ *bgs1Δ::ura4*$^{+}$ *leu1-32 ura4-Δ18 his3-Δ1*	This study
MBY 9085	h^{+}/h^{+} *sbg1Δ:kanMX/sbg1*$^{+}$ *ade6-M210/ade6-M216 ura4-D18/ura4-Δ18 leu1-32/leu1-32*	Bioneer Korea
MBY 9086	h^{+}/h^{-} *sbg1Δ:kanMX/sbg1*$^{+}$ *ade6-M210/ade6-M216 ura4-Δ18/ura4-Δ18 leu1-32/leu1-32*	This study
MBY 9097	*cps1-191 Hyg*^{r}*:eGFP-sbg1*$^{+}$ *ura4-Δ18 leu1-32* h^{-}	This study
MBY 9156	h^{+}/h^{-} *sbg1Δ:kanMX/sbg1*$^{+}$ *mCherry-atb2*$^{+}$*:hph rlc1*$^{+}$*-3GFP::kanMx ade6-M210/ade6-M216 ura4 -Δ18/ura4 -Δ18*	This study

MBY 9160	h$^+$/ h$^-$ *sbg1Δ:kanMX/sbg1$^+$ mCherry-atb2$^+$:hph bgs1Δ::ura4$^+$ P_{bgs1+}::GFP-bgs1 ade6-M210/ade6-M216*	This study
MBY 9188	*P_{bgs1+}::GFP-12A- cps1-191:leu1$^+$ bgs1Δ::ura4$^+$ leu1-32 ura4-Δ18 his3-Δ1 with pEmpty_his3* h$^-$	This study
MBY 9193	*P_{bgs1+}::GFP-12A- cps1-191: leu1$^+$ bgs1Δ:: ura4$^+$ leu1-32 ura4-Δ18 his3-Δ1 with pSbg1_his3* h$^-$	This study
MBY 9198	*his5Δ ura4-Δ18 leu1-32 ade5Δ ade7Δ::ade5$^+$ sbg1:his5cd:ura4$^+$* h$^-$	This study
MBY 9241	*Hyg^r:eGFP-sbg1$^+$ leu1-32 ura4-Δ18 bgs1Δ::ura4$^+$ P_{bgs1+}:3XHA-bgs1$^+$:leu1$^+$* h$^-$	This study
MBY 9285	*cps1-191 ura4-Δ18 leu1-32 his3-Δ1 with pmCherry-Sbg1*	This study
MBY 9358	*sbg1-3:his5$^+$:ura4$^+$ clp1Δ::ura4 leu1-32 ura4-Δ18 his3-Δ1 his5Δ ade5Δ ade7Δ::ade5$^+$* h$^-$	This study
MBY 9359	*sbg1-3:his5$^+$:ura4$^+$ his5Δ ura4-Δ18 leu1-32 ade5Δ ade7Δ::ade5$^+$* h$^-$	This study
MBY 9366	*sbg1-3:his5$^+$:ura4$^+$ his5Δ ura4-Δ18 leu1-32 ade5Δ ade7Δ::ade5$^+$ with pBgs1* h$^-$	This study
MBY 9370	*sbg1-3:his5$^+$:ura4$^+$ his5Δ ura4-Δ18 leu1-32 ade5Δ ade7Δ::ade5$^+$ with pSbg1* h$^-$	This study
MBY 9372	*sbg1-3:his5$^+$:ura4$^+$ his5Δ ura4-Δ18 leu1-32 ade5Δ ade7Δ::ade5$^+$ with pEmpty* h$^-$	This study
MBY 9389	*Hyg^r-eGFP-sbg1-3:his5$^+$:ura4$^+$ rlc1$^+$-tdTomato-NatMX6 pcp1$^+$-mCherry:ura4$^+$* h$^-$	This study
MBY 9390	*sbg1-3:his5$^+$:ura4$^+$ bgs1Δ::ura4$^+$ P_{bgs1+}::GFP-bgs1$^+$:leu1$^+$ rlc1$^+$-tdTomato-NatMX6 pcp1$^+$-mCherry:ura4$^+$* h$^+$	This study
MBY 9400	*sbg1-3:his5$^+$:ura4$^+$ imp2Δ::ura4+*	This study
MBY 9432	*Hyg^r-eGFP-sbg1$^+$ rlc1$^+$-tdTomato-NatMX6 pcp1$^+$-mCherry:ura4+* h$^+$	This study
MBY 9448	*sbg1-3:his5$^+$:ura4$^+$ pxl1Δ::KanMX6 GFP-pxl1$^+$:leu1$^+$ rlc1$^+$-tdtomato-NatMX6 pcp1$^+$-mcherry:ura4$^+$ leu1-32 ura4-Δ18* h$^-$	This study
MBY 9454	*cps1-191 rlc1$^+$-gfp:ura$^+$ pcp1$^+$-gfp:KanMX6 with pEmpty* h$^+$	This study
MBY 9456	*cps1-191 rlc1$^+$-gfp:ura$^+$ pcp1$^+$-gfp:KanMX6 with pSbg1* h$^+$	This study
MBY 9472	*cps1-191 ade6-M21x ura4-Δ18 leu1-32 with pSbg1-TMΔ* h$^+$	This study
MBY 9485	*sbg1-3:his5$^+$:ura4$^+$ rga7$^+$-GFP:KanMX6 rlc1$^+$-tdTomato-NatMX6 pcp1$^+$-mCherry::ura4$^+$* h$^-$	This study
MBY 9493	*rlc1$^+$-gfp:ura$^+$ pcp1$^+$-gfp:KanMX6 with pEmpty* h$^-$	This study
MBY 9508	*rlc1$^+$-gfp:ura$^+$ pcp1$^+$-gfp:KanMX6 with pSbg1* h$^-$	This study
MBY 11106	h$^+$/ h$^-$ *sbg1Δ:kanMX/sbg1$^+$ bgs1Δ::ura4$^+$ P_{bgs1+}::GFP-bgs1 ade6-M210/ade6-M216 ura4-Δ18/ ura4-Δ18 leu1-32/leu1-32*	This study
