# Supplementary material for: A New Membrane Protein Sbg1 Links the Contractile Ring Apparatus and Septum Synthesis Machinery in Fission Yeast
Source: PLoS Genet. 2016 Oct 17;12(10):e1006383. doi: 10.1371/journal.pgen.1006383 (PMC5066963; doi:10.1371/journal.pgen.1006383)
Supplement: S4 Table — (PDF) [file pgen.1006383.s008.pdf]

Table S4: Yeast strains used for two hybrid analysis

SGY37	*MATa leu2 ADE2 ura3-52::URA3-lexA-op-LacZ his3 trp1*	Geissler et al. [6]
YPH500	*MATα ura3-52 lys2-801 ade2-101 trp1Δ63 his3Δ200 leu2Δ1*	Sikorski et al. [7]
