# Supplementary material for: A New Membrane Protein Sbg1 Links the Contractile Ring Apparatus and Septum Synthesis Machinery in Fission Yeast
Source: PLoS Genet. 2016 Oct 17;12(10):e1006383. doi: 10.1371/journal.pgen.1006383 (PMC5066963; doi:10.1371/journal.pgen.1006383)
Supplement: S5 Table — (PDF) [file pgen.1006383.s009.pdf]

Table S5: Plasmids used in this study

pCDL1522	p*Hyg*^{r}*-eGFP:Amp*^{r}
pCDL1000	p*AL-KS-empty* (referred to as pEmpty)
pCDL1542	p*his5cd:ura4*$^{+}$
pCDL1456	p*his5c*$^{+}$
pCDL1673	p*sbg1*$^{+}$*:his5c*$^{+}$
pCDL1687	p*FA6a-mCherry-NatMX6*
pBac5	p*AL-KS-sbg1*$^{+}$ (referred to as pSbg1)
pBac14	p*AL-KS-sbg1-TMΔ* (referred to as pSbg1-TMΔ)
pBac15	p*AL-KS-bgs1*$^{+}$ (referred to as pBgs1)
pBac6	p*AL-KS-empty:his3*$^{+}$ (referred to as pEmpty_his3)
pBac7	p*AL-KS-sbg1*$^{+}$*:his3*$^{+}$ (referred to as pSbg1_his3)
pBac9	p*AL-KS-mCherry-sbg1*$^{+}$*:his3*$^{+}$
pMM5	pRS423-*Gal1-LexA-MYC*
pMM6	pRS425-*Gal1-Gal4-HA*
