# Supplementary material for: A New Membrane Protein Sbg1 Links the Contractile Ring Apparatus and Septum Synthesis Machinery in Fission Yeast
Source: PLoS Genet. 2016 Oct 17;12(10):e1006383. doi: 10.1371/journal.pgen.1006383 (PMC5066963; doi:10.1371/journal.pgen.1006383)
Supplement: S6 Table — (PDF) [file pgen.1006383.s010.pdf]

Table S6: Primers

MOH 1207	AATTAACCCTCACTAAAGGG
MOH 1208	GTAATACGACTCACTATAGGGC
MOH 6061	TATACGCGACATGGCGCGCG
MOH 6062	CGGTGAGTTCAGGCTTTTTCATTTTTTCTAATTAGTACTCTGCCTC
MOH 6063	GAGGCAGAGTACTAATTAGAAAAAATGAAAAAGCCTGAACTCACCG
MOH 6064	AGTCTATAGGTGACTGAGACATCTTGTACAGCTCGTCCATGCCGA
MOH 6065	TCGGCATGGACGAGCTGTACAAGATGTCTCAGTCACCTATAGACT
MOH 6066	TTTCTTCCTTTGCTTTTCTACTT
K150	GATAATGAATTGAATTACGAGTTGGCTAACGAAGTTTCA
K151	TTTCTACTTCCAAAATATTTACAAATCCACGCAAAGTAAAA
K152	AAATATTTTGGAAGTAGAAAAGCAAAGGAAGAAAAACTGA
K153	GGTCATTAGTTAAATGACTCGTATTTGCGAAA
K47	GGATGGTCGACGTTCAATCTCTAAATCTTTCA
K48	ATCCACAACTCAGCTGAGAACGGAGCTTACTCATCCAA
K49	GCTCCGTTCTCAGCTGAGTTGTGGATTGTTAATGCTTT

K50	AAGCACAACTAGTTGAAGGCAGTCG
K140	TACATTATACGAAGTCGACAGCTGAATTCTCGAGTGCTAGCGGATCCCGATATCAGGTGATTGGTATTTTATTTCGGCTGA
K141	GTTTTGTTCACTTATTTAAATAGATCTATAACTTCGTATAATGTATGCTATACGATTTGAAGGCAGTCGGTTAGACGAAAT
K154	AGAGTACTAATTAGAAAAAAATGGTGAGCAAGGGCGAGGA
K155	TCTATAGGTGACTGAGACATCTTGTACAGCTCGTCCATGCCG
K185	GACGAAGCTCTTTCTAGAAGCGTAGT
K186	GGTGATTGGTATTTTATTTCGGCTGA
