# Supplementary material for: A New Membrane Protein Sbg1 Links the Contractile Ring Apparatus and Septum Synthesis Machinery in Fission Yeast
Source: PLoS Genet. 2016 Oct 17;12(10):e1006383. doi: 10.1371/journal.pgen.1006383 (PMC5066963; doi:10.1371/journal.pgen.1006383)
Supplement: S1 Text — (DOCX) [file pgen.1006383.s011.docx]

**Supplementary data**

**Materials and methods**

**Membrane association experiments**

Early-log phase S. pombe cells were harvested and lysed using glass beads in lysis buffer (50 mM Tris-HCl, pH 7.6, 150 mM NaCl, 1 mM EDTA, 10% glycerol) containing protease inhibitors (Complete EDTA-free; Roche Diagnostics, Basel, Switzerland, 2 mM PMSF, 2 mM Benzamidine). Buffers to test the association of Sbg1p with the plasma membrane were based on published literature [1,2]. Briefly, cells were treated with buffer alone, or buffer with 1.6M urea or 5M NaCl (solubilizes peripheral membrane proteins), 0.1 M Na_2_CO_3_ (solubilizes intracellular vesicles), 4% Triton-X 100 (solubilizes most membrane proteins), or 2% SDS (solubilizes all membrane proteins). We also analysed solubility of the proteins in 0.5% Tween-20 and 0.8% Digitonin. Lysates were subjected to ultracentrifugation at 100,000 X *g* for 1 hr at 4°C. Supernatants and pellets were separated and boiled in sample buffer. SDS-PAGE and immunoblotting conditions were followed as described in the previous section for immunoprecipitation experiments.

**Labelling and fractionation of cell wall polysaccharides**

Cell wall analysis was performed as described previously [3,4]. Exponentially growing cells at 24ºC in minimal medium lacking leucine were diluted and supplemented with D-[^14^C]-glucose (3 µCi/ml), maintained at 24ºC for 24 hr or shifted to 34ºC for 16 hr (see S1 Table). For analysis of *sbg1-3* mutant and comparison to *cps1-191* at 36ºC, *sbg1-3* cells were grown at 24ºC or shifted to 36ºC for 24 hr for expression of a significant *sbg1-3* mutant phenotype and for *cps1-191,* the shift up was for 4.5 hr for expression of the allowed maximal phenotype before cell death appeared (see S2 Table)*,* D-[^14^C]-glucose (10 µCi/ml) was added 2.5 hr before harvesting the cells at 36ºC to only label the cell wall with maximum defects. Harvested cells were supplemented with unlabeled cells as carrier, washed twice with 1 mM EDTA, and resuspended in 1 mM EDTA. Two aliquots of cells were added to liquid scintillation cocktail and total D-[^14^C]-glucose incorporation in the cells was assessed from this. Cell walls were purified from bead-beaten lysed cells by repeated washing and differential centrifugation (once with 1 mM EDTA, twice with 5 M NaCl, and three times with 1 mM EDTA) at 1,500 g for 5 min. Purified cell walls were heated at 95°C for 30 min and D-[^14^C]-glucose incorporation in the cell walls was monitored in two aliquots. One part of cell wall samples was extracted with 6% NaOH for 60 min at 80°C. The galactomannan fraction was precipitated from the supernatant with Fehling´s reagent by adding unlabeled yeast mannan (4 mg) as the carrier, as described previously [5]. Four volumes of Fehling’s reagent were added to the sample and allowed to precipitate galactomannan overnight at 4°C. Pellets were obtained by centrifugation at 4,000 g for 10 min, washed with Fehling’s reagent and solubilized in 6N HCl. The galactomannan fraction was determined from this after addition of 50 mM Tris-HCl, pH 7.5 by measuring the radioactivity in a scintillation counter (Perkin Elmer). Second part of cell wall suspensions was incubated with Zymolyase 100T (AMS Biotechnology; MP Biomedicals) in 50 mM citrate-phosphate buffer (pH 5.6) for 24 h at 37°C, using untreated samples as control. Pellets from centrifugation were resuspended in 1 mM EDTA. Liquid scintillation cocktail was added to this and radioactivity levels were measured with pellets corresponding to cell wall α-glucan fraction and supernatants to β-glucan-plus-galactomannan fraction. Third part of cell wall suspension was incubated with Quantazyme (MP Biomedicals; Q-Biogene) in 50 mM potassium phosphate monobasic (pH 7.5), 60 mM β-mercaptoethanol for 24 h at 37°C. Radioactivity of pellets was measured after centrifugation and this corresponded to cell wall without β-1,3-glucan fraction, while supernatant was considered as β-1,3-glucan fraction. β-1,6-glucan was calculated as the remaining polysaccharide from total cell wall radioactivity minus radioactivity of galactomannan, α-glucan and β-1,3-glucan. All determinations were performed in duplicates, with at least three independent replicates for each strain (five independent experiments for the analysis of wild type and *cps1-191* at 24ºC and 34ºC, four independent experiments for the analysis of wild type and *sbg1-3* at 36ºC, and three independent experiments for the analysis of wild type and *cps1-191* at 36ºC).

**Supplementary references:**

1. Harkins H a, Pagé N, Schenkman LR, De Virgilio C, Shaw S, Bussey H, et al. Bud8p and Bud9p, proteins that may mark the sites for bipolar budding in yeast. Mol Biol Cell. 2001;12: 2497–518.

2. Liu J, Wang H, Mccollum D, Balasubramanian MK. Drc1p/Cps1p, a (1,3)b-glucan Synthase Subunit, Is Essential for Division Septum Assembly in Schizosaccharomyces pombe. Genetics. 1999; 1193–1203.

3. Ishiguro J. Genetic control of fission yeast cell wall synthesis: the genes involved in wall biogenesis and their interactions in Schizosaccharomyces pombe. Genes Genet Syst. 1998;73: 181–191. doi:10.1266/ggs.73.181

4. Pérez P, Ribas JC. Cell wall analysis. Methods. 2004;33: 245–251. doi:10.1016/j.ymeth.2003.11.020

5. Algranati ID, Behrens N, Carminatti H, Cabib E. Complex Carbohydrates [Internet]. Methods in Enzymology. Elsevier; 1966. doi:10.1016/0076-6879(66)08075-3

6. Geissler S, Pereira G, Spang A, Knop M, Souès S, Kilmartin J, et al. The spindle pole body component Spc98p interacts with the gamma-tubulin-like Tub4p of Saccharomyces cerevisiae at the sites of microtubule attachment. EMBO J. 1996;15: 3899–911.

7. Sikorski RS, Hieter P. A system of shuttle vectors and yeast host strains designed for efficient manipulation of DNA in Saccharomyces cerevisiae. Genetics. 1989;122: 19–27.
